# Repeated Introductions and Anthropogenic Dispersal Fuel the Regional Expansion of *Ambrosia trifida*

**DOI:** 10.1111/eva.70333

**Published:** 2026-10-21

**Authors:** Tae‐Young Choi, Dong‐Chan Son, Soo‐Rang Lee

**Affiliations:** ^1^ Division of Science Education, College of Education Jeonbuk National University Jeonju Republic of Korea; ^2^ Division of Forest Biodiversity and Herbarium Korea National Arboretum Pocheon Republic of Korea; ^3^ Department of Biology Education, College of Education Chosun University Gwangju Republic of Korea

**Keywords:** *Ambrosia*, giant ragweed, invasion history, invasive species, multiple introductions, niche comparisons

## Abstract

Understanding the processes that shape biological invasions is essential for predicting the spread and persistence of introduced species, yet these processes remain poorly resolved for many unintentionally introduced plants. We combined genome‐wide SNP data, demographic modeling, and climatic niche analyses to investigate the invasion of giant ragweed in South Korea. A total of 419 individuals from 35 introduced populations and 59 individuals from four native North American populations were analyzed. Introduced populations retained high genetic diversity and exhibited weak spatial structure, consistent with extensive human‐mediated dispersal. Approximate Bayesian Computation supported a recent invasion within the last ~70 years involving multiple introductions from genetically divergent sources, followed by rapid divergence and severe but transient demographic bottlenecks. Climatic niche analyses revealed substantial overlap between native and introduced ranges but pronounced niche unfilling, indicating that *Ambrosia trifida* has yet to occupy its full potential climatic niche in South Korea. Together, these results show that repeated introductions, admixture, and anthropogenic dispersal, rather than major climatic niche shifts, have shaped the contemporary invasion. Our findings highlight how integrating population genomics with niche analyses can resolve invasion processes and inform management of rapidly spreading invasive species.

## 1 Introduction

Biological invasions have increased markedly in recent centuries, largely as a consequence of intensified human activities (Vitousek et al. 1996; Pimentel et al. 2000; Mooney and Cleland 2001; Diagne et al. 2021; Seebens et al. 2021). Because invasive species establish and spread beyond their native ranges, invasions are accompanied by rapid demographic changes that generate spatial and temporal variation in genetic diversity. These demographic and genetic dynamics play a central role in shaping evolutionary potential and invasion success (Lee 2002; Prentis et al. 2008; Dlugosch et al. 2015; Estoup et al. 2016; Van Boheemen et al. 2017; Sherpa and Després 2021).

Early invasion stages are commonly characterized by population bottlenecks and founder effects, which reduce genetic diversity and may limit adaptive responses to novel environments (Frankham 1995; Blackburn et al. 2015). Nevertheless, many invasive species thrive despite such constraints, giving rise to the genetic paradox of invasion. This paradox can be resolved through genetic admixture, either via multiple introductions from genetically divergent source populations or through subsequent dispersal within the introduced range (Dlugosch and Parker 2008; Prentis et al. 2008; Lee and Son 2022; Lee et al. 2024). In some cases, an introduced population becomes the primary source for further spread through a bridgehead effect (Bertelsmeier and Keller 2018; Vallejo‐Marín et al. 2021). Consequently, reconstructing invasion history, including identification of source populations and dispersal pathways, is essential for understanding the demographic and evolutionary processes underlying invasion success. However, such reconstructions remain challenging because introductions are often poorly documented and historical records are incomplete (Estoup and Guillemaud 2010; Cristescu 2015; Vallejo‐Marín et al. 2021).

The recent surge of biological invasions has highlighted the value of population genetics for understanding why some species become successful invaders. Advances in genomic technologies have greatly expanded the power of population genetic analyses. Genomic data, together with robust analytical approaches, offer detailed insights into genetic diversity, adaptation, demographic history, source identification, dispersal routes, and hybridization (Cristescu 2015; McGaughran et al. 2024; Hodgins et al. 2025). For example, analysis of approximately 3500 SNPs from about 280 genotypes of *Hypochaeris radicata* revealed multiple introduction events followed by human‐mediated range expansion of this noxious weed (Lee et al. 2024). By examining genetic variation across contemporary populations, population genetic approaches can reveal previously hidden aspects of invasion history.

*Ambrosia trifida* L. (Asteraceae), commonly known as giant ragweed, is a short‐day annual herb recognized as one of the most destructive weeds in European and Asian crop fields (Schutte 2007; EPPO 2020; Kato‐Noguchi and Kato 2024). Though native to North America, this species has been introduced and now spans all northern temperate continents, extending its range as far north as 66° latitude (www.gbif.org; Chauvel et al. 2021). Throughout its range, the species most commonly occurs along roadsides, in cultivated fields and along water courses with rich, moist soils (Bassett and Crompton 1982; Regnier et al. 2016; EPPO 2020; Chauvel et al. 2021). *Ambrosia trifida* exhibits several biological traits crucial for successful invasion, including high fecundity, pronounced phenotypic plasticity, rapid growth, and resistance to various abiotic stresses, with the exception of drought (Wortman et al. 2012; Korres et al. 2015; EPPO 2020). The species' rapid growth and strong competitive ability facilitate the formation of dense, monospecific stands, which exert allelopathic effects on neighboring plants and pose substantial threats to ecosystem integrity and crop productivity (Abul and Bazzaz 1979; Washitani 2001; Wang et al. 2005; Lee et al. 2010; Regnier et al. 2016; EPPO 2020; Kato‐Noguchi and Kato 2024). Beyond its impacts on agriculture and biodiversity, giant ragweed poses substantial public health concerns as a major source of allergenic pollen, a principal cause of hay fever (Rasmussen et al. 2017; Chauvel et al. 2021). Given these negative impacts and its rapid spread rate, the species was already recognized as one of the top 20 worst weeds in the western states of the US as early as the late 19th century (Halsted 1889).

*Ambrosia trifida* primarily occupies latitudes between 30° and 45° North, a range limited by its strict flowering phenology (Allard 1943). The species began invading Europe in the late 19th century and has since become widespread across the continent, particularly in Western Europe (Chauvel et al. 2015; EPPO 2020). Approximately half a century later, *A. trifida* began spreading in East Asia, including Korea, China, Mongolia, and Japan (Qin et al. 2014; Lee and Oh 1974; Kim 2017; Kim and Kil 2016; EPPO 2020). Introductions to both Europe and Asia have largely been attributed to the inadvertent transport of *A. trifida* seeds through contaminated animal feed and crop seed lots, particularly soybean and maize (Follak et al. 2013; Chauvel et al. 2015; EPPO 2020). Although the plant's seeds primarily disperse a few meters by gravity, they can be transported over much longer distances via water, rodents, birds, and other animal vectors (Harrison et al. 2003; Regnier et al. 2008; Chauvel et al. 2021). Due to its high dispersal capacity and ecological competitiveness, *A. trifida* is now regarded as one of the most problematic invasive weeds in both Europe and Asia (Chauvel et al. 2021).

In Korea, *A. trifida* was first recorded in 1964 by a botanist in the northern part of the country, with several additional observations reported through the 1980s (Kim et al. 2000; Lee and Oh 1974; Lee and Chong 1976; Kim and Kil 2016). Although direct historical evidence is limited, previous studies have suggested that the species was introduced to Korea during the 1970s through contaminated animal feed and supplies associated with the US military (Kil et al. 2004; Kim and Kil 2016). A rapid spread followed in the 1980s, likely facilitated by extensive development activities linked to the country's economic boom. In 1999, *A. trifida* was officially designated as a harmful invasive alien species requiring government control (Kim and Kil 2016; Cho et al. 2022). Today, giant ragweed is one of the most prevalent invasive weeds across South Korea. It is especially abundant in riparian systems, where it colonizes riverbanks, waterways, wetlands, and service roads, posing significant threats to native riparian ecosystems (Lee et al. 2010; Cho et al. 2022).

Recent population genomic studies of the closely related common ragweed ( *Ambrosia artemisiifolia* ) have greatly advanced our understanding of these dynamics, providing a valuable comparative framework for understanding invasion genetics within the genus *Ambrosia*. Genome‐wide SNP dataset combined with demographic modeling has clarified invasion routes, quantified post‐introduction admixture, and identified the demographic processes underlying invasion success in *A. artemisiifolia* (Genton et al. 2005; Chun et al. 2009; Van Boheemen et al. 2017; Gorton et al. 2018; Li et al. 2019; Prapas et al. 2022; Battlay et al. 2025). Given that *A. trifida* shares many ecological and life‐history traits with its congener, including an annual life cycle, wind‐pollination, and obligate outcrossing, these studies provide a useful comparative framework for interpreting invasion processes in giant ragweed. However, population genomic investigations for *A. trifida* remain scarce, leaving its invasion processes in East Asia poorly resolved.

To address this knowledge gap, we investigated the invasion processes underlying the successful establishment and spread of *A. trifida* in South Korea using genome‐wide SNP data. Our dataset comprised 419 individuals representing 35 introduced populations and 59 individuals from four native populations in the United States. We characterized patterns of genetic diversity and population structure, inferred demographic history and introduction scenarios using an Approximate Bayesian Computation (ABC) framework, and evaluated climatic niche dynamics between the native and introduced ranges.

## 2 Materials and Methods

### 2.1 Study System, Sample Collection & DNA Extraction

Giant ragweed ( *A. trifida* ) is a tall annual plant that can reach up to 6 m in height under favorable conditions. The species is predominantly outcrossing due to its monoecious floral arrangement, in which male and female flowers are separated on the same plant, with female flowers emerging several days after the onset of male flowering (Bazzaz and Carlson 1979; Chauvel et al. 2021; Kato‐Noguchi and Kato 2024). Flowering typically begins in August and continues until the first killing frost. Each plant can produce approximately 2000 seeds, over 50% of which are viable. However, seed production varies widely among populations (Bazzaz and Carlson 1979; Harrison et al. 2001; Goplen et al. 2016). Pollination is wind‐mediated (Bassett and Crompton 1982). Seed dispersal occurs mainly via water and animal‐mediated transport although longer‐distance dispersal may occur through rodents, birds, and human activities (Harrison et al. 2003). Seeds break dormancy following exposure to moist, cool conditions for approximately 3 months (Davis 1930) and can remain viable for 4–20 years, depending on burial depth (Stoller and Wax 1974; Chauvel et al. 2021).

Sampling was conducted during the summers of 2022 and 2024, yielding a total of 558 individual samples from 39 populations. Our sampling scheme included four native populations and 35 introduced populations (Table 1; Figure 1a). For the introduced region, we randomly selected sample sites across the entire country, ensuring a minimum distance of 50 km between populations. To minimize sampling of closely related individuals, we maintained a collection distance of at least 30 m between plants within each population. Because the native range spans much of North America, sampling was limited to four populations in the North Central US, which were separated by a minimum of 30 km. All fresh leaf tissues were immediately preserved in silica gel for DNA extraction.

**TABLE 1:** Summary of genetic diversity indices estimated based on 3504 SNPs and sampling sites for *Ambrosia trifida* .

Location	Population acronym	Cluster assignment	No. of samples	GPS coordinates		*H*_o_	H_*e*_	*N*_a__rare
				*X*	*Y*			
Asan‐si, Chungcheongnam‐do, South Korea	AS	Deme 1	15	36.795331	127.086016	0.23 [0.16]	0.30 [0.15]	1.70 [0.33]
Bonghwa‐gun, Gyeongsangbuk‐do, South Korea	BD	Excluded	14	37.001285	128.812628	0.25 [0.18]	0.27 [0.16]	1.60 [0.40]
Gijang‐gun, Busan, South Korea	BF	Excluded	15	35.309412	129.241015	0.18 [0.15]	0.24 [0.15]	1.67 [0.34]
Buyeo‐gun, Chungcheongnam‐do, South Korea	BY	Deme 1	14	36.282612	126.898861	0.16 [0.14]	0.24 [0.16]	1.63 [0.37]
Dongducheon‐si, Gyeonggi‐do, South Korea	DD	Deme 1	15	37.89577	127.03755	0.17 [0.18]	0.18 [0.13]	1.77 [0.37]
Dalseo‐gu, Daegu, South Korea	DG	Deme 1	6	35.81872	128.48879	0.26 [0.19]	0.33 [0.14]	1.59 [0.47]
Yuseong‐gu, Daejeon, South Korea	DJ	Deme 1	15	36.375789	127.377422	0.19 [0.17]	0.22 [0.14]	1.61 [0.36]
Buk‐gu, Busan, South Korea	GB	Deme 2	12	35.226083	129.006383	0.24 [0.17]	0.28 [0.15]	1.70 [0.42]
Goseong‐gun, Gangwon‐do, South Korea	GF	Deme 1	15	38.51922	128.4016	0.26 [0.20]	0.27 [0.17]	1.51 [0.43]
Gyeongju‐si, Gyeongsangbuk‐do, South Korea	GJ	Excluded	14	35.926946	129.242523	0.26 [0.17]	0.29 [0.14]	1.70 [0.32]
Gangneung‐si, Gangwon‐do, South Korea	GR	Deme 2	10	37.76563	128.91724	0.24 [0.18]	0.27 [0.15]	1.74 [0.42]
Goseong‐gun, Gangwon‐do, South Korea	GS	Deme 1	10	38.38775	128.4607	0.26 [0.18]	0.30 [0.15]	1.61 [0.41]
Hanam‐si, Gyeonggi‐do, South Korea	HN	Deme 1	15	37.747242	127.188347	0.22 [0.15]	0.29 [0.14]	1.73 [0.28]
Hwaseong‐si, Gyeonggi‐do, South Korea	HS	Deme 1	4	37.211852	126.735364	0.29 [0.21]	0.39 [0.12]	1.80 [0.49]
Seo‐gu, Incheon, South Korea	IC	Deme 1	11	37.527492	126.653068	0.23 [0.15]	0.30 [0.14]	1.72 [0.31]
Inje‐gun, Gangwon‐do, South Korea	IF	Deme 1	15	37.98724	128.1011	0.20 [0.17]	0.25 [0.15]	1.61 [0.37]
Imsil‐gun, Jeonbuk‐do, South Korea	IM	Excluded	12	35.692641	127.269586	0.25 [0.19]	0.26 [0.16]	1.54 [0.43]
Inje‐gun, Gangwon‐do, South Korea	IO	Deme 1	7	38.22207	128.36822	0.28 [0.18]	0.31 [0.14]	1.76 [0.42]
Inje‐gun, Gangwon‐do, South Korea	IS	Deme 1	9	37.95588	128.3716987	0.28 [0.21]	0.29 [0.15]	1.94 [0.45]
Inje‐gun, Gangwon‐do, South Korea	IT	Deme 1	5	38.24677	128.20933	0.30 [0.20]	0.35 [0.13]	1.61 [0.47]
Gyeongsan‐si, Gyeongsangbuk‐do, South Korea	KS	Excluded	14	35.789842	128.722992	0.25 [0.17]	0.32 [0.16]	1.67 [0.35]
Chuncheon‐si, Gangwon‐do, South Korea	MC	Deme 1	13	37.821528	127.706037	0.24 [0.17]	0.29 [0.14]	1.71 [0.32]
Mungyeong‐si, Gyeongsangbuk‐do, South Korea	MG	Excluded	14	36.634417	128.200208	0.23 [0.19]	0.27 [0.16]	1.65 [0.35]
Samcheok‐si, Gangwon‐do, South Korea	MI	Excluded	13	37.42105	129.11729	0.23 [0.18]	0.28 [0.14]	1.68 [0.35]
Miryang‐si, Gyeongsangnam‐do, South Korea	MY	Deme 3	14	35.485635	128.727491	0.26 [0.20]	0.27 [0.15]	1.64 [0.36]
Paju‐si, Gyeonggi‐do, South Korea	PF	Deme 1	5	37.781264	126.695636	0.27 [0.19]	0.36 [0.13]	1.61 [0.47]
Paju‐si, Gyeonggi‐do, South Korea	PS	Deme 1	5	37.891819	126.814462	0.30 [0.21]	0.35 [0.13]	1.63 [0.46]
Sinan‐gun, Jeollanam‐do, South Korea	SA	Deme 1	13	34.751622	126.145344	0.25 [0.17]	0.31 [0.15]	1.66 [0.37]
Sokcho‐si, Gangwon‐do, South Korea	SC	Deme 1	13	38.22189	128.58103	0.24 [0.18]	0.26 [0.16]	1.67 [0.35]
Suwon‐si, Gyeonggi‐do, South Korea	SW	Deme 1	14	37.249589	127.041452	0.24 [0.17]	0.31 [0.15]	1.75 [0.29]
Ulju‐gun, Ulsan, South Korea	US	Excluded	14	35.581417	129.23619	0.25 [0.16]	0.31 [0.15]	1.72 [0.31]
Yeongcheon‐si, Gyeongsangbuk‐do, South Korea	YC	Excluded	9	35.980681	128.9001619	0.23 [0.16]	0.31 [0.14]	1.68 [0.42]
Yeoncheon‐gun, Gyeonggi‐do, South Korea	YE	Deme 1	13	38.083482	127.076803	0.23 [0.16]	0.28 [0.14]	1.68 [0.34]
Yeoju‐si, Gyeonggi‐do, South Korea	YJ	Deme 1	22	37.297738	127.651744	0.21 [0.12]	0.29 [0.13]	1.68 [0.31]
Yeongwol‐gun, Gangwon‐do, South Korea	YW	Deme 1	10	37.224271	128.334593	0.22 [0.17]	0.27 [0.14]	1.65 [0.38]
Dunn Ranch Prairie, MO, USA	Native 1		15	40.500480	−94.099562	0.21 [0.15]	0.26 [0.15]	1.70 [0.30]
Prairie State Park, MO, USA	Native 2		14	37.517651	−94.553471	0.16 [0.14]	0.23 [0.14]	1.75 [0.26]
Nachusa Grasslands, IL, USA	Native 3		15	41.883772	−89.340002	0.20 [0.15]	0.26 [0.14]	1.75 [0.26]
Omaha Prairie, NE, USA	Native 4		15	41.230349	−96.094502	0.18 [0.15]	0.22 [0.15]	1.71 [0.30]

*Note:* The cluster assignment corresponds to the redefined groups used in the ABC analysis. *H*_o_, *H*_e_, and *N*_a__rare refer to observed heterozygosity, expected heterozygosity, and the number of alleles adjusted for population sample sizes, respectively. Numbers in brackets indicate standard errors.

**FIGURE 1:**
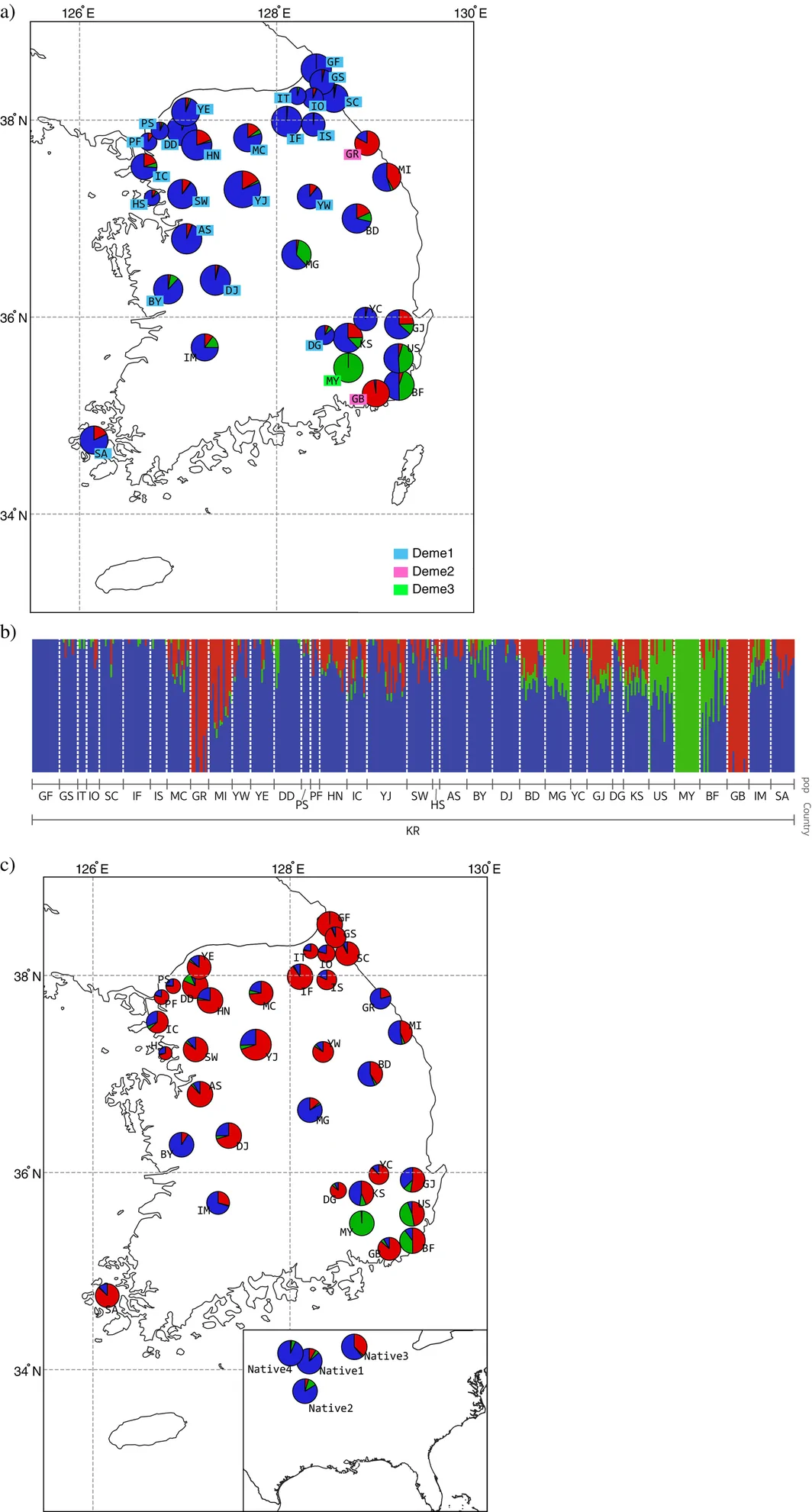
Genetic structure and geographic distribution of 39 *Ambrosia trifida* populations from the native (US; *n* = 4) and introduced (South Korea; *n* = 35) ranges. (a) Spatial distribution of genetic clusters in South Korea inferred from fastSTRUCTURE at *K* = 3. Pie charts show mean ancestry proportions per population, with sizes proportional to sample size. Populations are color coded and labeled according to their membership in the two primary genetic clusters (Demes 1 and 2) identified by STRUCTURE and PCoA analysis. (b) Individual Bayesian clustering results for 419 individuals at *K* = 3. Each vertical bar represents an individual, and colors indicate proportional membership in the three inferred genetic clusters. Populations are separated by dashed white lines and labeled according to Table 1. (c) Global distribution of genetic clusters across native and introduced populations, shown as cumulative ancestry proportions per population at *K* = 3.

Genomic DNA was extracted from dried leaf tissue using the DNeasy Plant Mini Kit (Qiagen, Hilden, Germany) following the manufacturer's protocol. DNA quality was assessed by electrophoresis on a 1% agarose gel. DNA concentration was measured using a Qubit 4 Fluorometer (Thermo Fisher Scientific, MA), and the extracted DNA was stored at −20°C.

### 2.2 RAD‐Seq Library Preparation & Raw Data Processing

Genomic DNA samples were genotyped using the 2RAD method (Bayona‐Vásquez et al. 2019), a modified version of the widely used reduced‐representation sequencing technique ddRAD (Peterson et al. 2012). Library preparation followed the protocol outlined in Bayona‐Vásquez et al. (2019), with minor modifications. Genomic DNA was digested using a frequent cutter, EcoRI‐HF (Thermo Fisher Scientific), and a rare cutter, XbaI (Thermo Fisher Scientific). Following digestion, adapter ligation, pooling, and cleanup were performed. We then carried out a size selection to target 450–500 bp fragments using a Pippin Prep system (Sage Science, Beverly, MA). PCR amplification was performed, followed by cleanup of the PCR products. All purification steps throughout the protocol used a 1:1.8 ratio of AMPure XP beads, followed by a 70% ethanol wash. Final library quality was assessed with an Agilent 2100 Bioanalyzer (Agilent Technologies, Santa Clara, CA, USA). Libraries were subsequently sequenced on an Illumina NovaSeq platform at Macrogen Inc. (Seoul, Korea) using 2 × 150 bp paired‐end reads.

Raw sequence reads were demultiplexed, quality filtered (Phred score threshold = 10; sliding window = 0.15), and trimmed to 141 bp using the process_radtags module in Stacks v. 2.41 (Rochette et al. 2019). The polished reads were subsequently mapped to the *A. trifida* genome (GCA_028751855.1) using Bowtie v. 2.2.3 (Langmead and Salzberg 2012), retaining only reads with a mapping quality (MAPQ) of 30 or higher. We employed the ref_map.pl. module in Stacks to identify and assemble RAD loci from the aligned reads, using a minimum stack depth of three (‐m3). During catalog construction, up to two mismatches between loci were allowed (cstacks ‐n 2). SNPs were called using the populations module, requiring loci to be present in at least 15 populations (‐p 15) and in at least 80% of individuals within each population (‐r 0.8). To minimize the influence of physical linkage among markers, only the first SNP from each RAD locus was retained for downstream analyses. To ensure a high‐quality SNP dataset, we applied several additional filtering steps. First, we removed SNPs that deviated significantly from Hardy–Weinberg equilibrium (*p* < 10^−6^) using Plink v. 1.9 (Purcell et al. 2007), thereby reducing the potential influence of genotyping errors, paralogous loci and assembly artifacts. Finally, genotypes with excessive missing data were removed using Tassel v. 5.0 (Glaubitz et al. 2014), and SNPs with a minor allele frequency (MAF ≤ 0.1) were excluded to reduce the influence of rare alleles that are more susceptible to genotyping error and contribute little to inference of broad‐scale population structure. A slightly more conservative MAF threshold than the commonly used value of 0.05 was adopted because the preliminary dataset contained numerous low‐frequency variants restricted to single or very few populations. Such rare alleles substantially increased background noise in multivariate and clustering analyses while providing limited information for reconstructing large‐scale invasion history and demographic relationships, which were the primary objectives of this study. These filtering criteria yielded a final dataset of 3504 high‐quality, unlinked SNPs for downstream population genomic analyses.

### 2.3 Genetic Diversity & Population Structure

We used Arlequin v.3.5 (Excoffier and Lischer 2010) to compute observed (*H*_o_) and expected (*H*_e_) heterozygosities for each population from the final dataset of 3504 SNPs. To account for unequal sample sizes, the number of alleles (*N*_a_) was calculated using rarefaction curves in HP‐Rare (Kalinowski 2005) (Table 1; Kalinowski 2004). Population‐level estimates of *H*_e_, *H*_o_, and *N*_a_ were then compared between the four native populations and the 35 introduced populations using two‐tailed Welch's *t*‐tests, which do not assume equal variances, in Microsoft Excel. To assess the extent of population divergence, we estimated pairwise *F*_ST_ values for all 39 populations in Arlequin, with statistical significance determined by 1000 permutations. In addition, we tested for Isolation By Distance (IBD) within the introduced range to evaluate whether Korean populations (35 populations) have reached migration–drift equilibrium (Briscoe Runquist and Moeller 2024). IBD was assessed using a Mantel test examining the correlation between linearized pairwise genetic differentiation (*F*_ST_/(1 − *F*_ST_)) and Euclidean geographic distances (Rousset 1997), as implemented in GenAlEx v. 6.51 (Peakall and Smouse 2012). Statistical significance was evaluated using 1000 random permutations.

We investigated the population genetic structure of 39 populations using 3504 unlinked SNPs through two complementary approaches. First, principal component analysis (PCA) was conducted based on Nei's genetic distance calculated from 478 individuals using GenAlEx. Second, a Bayesian clustering analysis was performed using fastSTRUCTURE (Raj et al. 2014) to infer the number of genetic clusters (*K*) and individual admixture proportions. This program is optimized for large‐scale genomic data, making it a suitable alternative to the original STRUCTURE software (Pritchard et al. 2000). We tested a range of *K* values from 1 to 12. Each *K* run was replicated 20 times to ensure consistency of the inferred ancestry coefficients. The analysis was performed using the “logistic” prior, which is well‐suited for populations with varying sizes and complex structures. The optimal *K*, representing the most likely number of randomly mating clusters, was determined using the chooseK.py utility script distributed with the fastSTRUCTURE package, which estimates the model complexity that maximizes the marginal likelihood while avoiding unnecessary over‐partitioning of the data (Raj et al. 2014). Individual ancestry coefficients for the selected *K* were visualized using the accompanying distruct.py script. To investigate finer‐scale genetic structure within the introduced range, fastSTRUCTURE was also performed on a subset containing only the 35 introduced Korean populations. This analysis was conducted separately because the pronounced genetic differentiation between the native US and introduced Korean populations could mask more subtle population structure within Korea. The Korean‐only analysis was therefore intended to resolve within‐range genetic structure rather than to directly compare *K* values with those inferred from the full dataset.

Given the complex clustering patterns and broad range of optimal *K*, we further assessed population splits and historical migration patterns using TreeMix v. 1.13 (Pickrell and Pritchard 2012). This method estimates a maximum‐likelihood population tree from allele frequencies based on shared genetic drift, and subsequently adds migration events to account for exceptional shared drift. To reduce complexity, seven highly admixed populations were excluded from this analysis (Table 1), with linkage among loci was minimized by retaining only one SNP per RAD locus during SNP filtering. We inferred a maximum‐likelihood phylogenetic tree for the three major demes identified from fastSTRUCTURE, rooting the tree with the four native US populations and 10 samples of *A. artemisiifolia* . The covariance matrix was estimated with 500 bootstrap replicates and a SNP block size of 100 (‐k). We tested a range of migration events from 0 to 5, and the optimal number was determined using the R package OptM (Fitak 2021).

To assess the robustness of our population genetic inferences, we conducted two complementary sensitivity analyses. First, to evaluate the potential influence of unequal sample sizes between native (*n* = 59) and introduced (*n* = 419) samples, we randomly subsampled 59 individuals from the introduced Korean dataset to match the native sample size. This procedure was repeated independently 10 times. For each replicate, PCoA and fastSTRUCTURE analyses were performed using the same parameters as for the full dataset. Because the downsampled datasets consisted of randomly selected individuals from across Korea, local population assignments were not retained and all Korean individuals were treated as a single introduced group. The resulting clustering patterns and inferred values of *K* were compared with those obtained from the full dataset. Second, to evaluate the potential effect of SNP filtering on the representation of native‐range variation, we applied the minor allele frequency (MAF) threshold separately to the native US samples while retaining the original filtering procedure for the introduced samples. Genetic diversity statistics, PCoA, and fastSTRUCTURE analyses were then repeated using the alternatively filtered dataset and compared with those obtained from the original global MAF‐filtered dataset.

### 2.4 Niche Analysis

We evaluated niche shifts in the introduced region relative to the native range using the ordination‐based PCA‐env approach, which has been shown to outperform other niche modeling methods (Broennimann et al. 2012; Petitpierre et al. 2012; Guisan et al. 2014). Georeferenced occurrence data for the native range were obtained from the Global Biodiversity Information Facility (GBIF), yielding 36,400 records. The extent of the native range was defined following EPPO (2020). For Korea, we compiled 2365 records by combining GBIF data with additional sources, including the Korean National Species Information System (https://www.nature.go.kr) and the National Survey on the Natural Environment (https://www.data.go.kr/data).

We extracted 19 climatic variables at a 30‐arcsecond resolution from WorldClim v2.1 (http://www.worldclim.org/; Fick and Hijmans 2017). All environmental layers were cropped to the study area and converted to a common raster format in ArcGIS v10.6 prior to PCA‐env analyses. PCA‐env analyses were conducted using the R package “ecospat” (Broennimann et al. 2012; Di Cola et al. 2017; R Core Team 2025). Niche shifts were quantified in terms of overlap (D), unfilling (U), and expansion (E) to assess post‐invasion niche change (Godsoe 2010; Broennimann et al. 2012; Guisan et al. 2014; Liu et al. 2020). Schoener's *D* and Warren's *I* indices (Warren et al. 2008) were calculated using the R package ENMtools v1.0.4 (Warren et al. 2010; R Core Team 2025). Niche equivalency and similarity tests (Warren et al. 2008) were performed with 100 iterations to assess the statistical significance of niche overlap.

### 2.5 Inferring Invasion History

We employed an ABC framework to infer the demographic history of *A. trifida* (giant ragweed) invasion in Korea using DIYABC Random Forest (DIYABC‐RF) version 1.0 (Collin et al. 2021). Because the ABC framework evaluates alternative demographic scenarios among predefined, discrete population lineages rather than continuous patterns of admixture, we grouped samples into three clusters (Demes 1–3) based on the combined results of fastSTRUCTURE and TreeMix (Table 1). To meet this assumption, seven highly admixed populations, defined as those with a mean ancestry coefficient < 0.7 for any single genetic cluster (Figure 1a), were excluded because they could not be reliably assigned to a single demographic lineage. Additional populations with small sample sizes were also excluded, resulting in a total of 12 populations removed from the ABC analyses. Given that our sampling from the native range was restricted to the North Central United States and was limited in size, the native samples may not adequately represent the full extent of genetic diversity across the species' native range. To account for this limitation, we included two unsampled “ghost populations” (populations 5 and 6), representing potential but uncollected genetic sources from the native area that may have contributed to introductions in Korea (Cornuet et al. 2010).

Based on the inferred genetic structure, TreeMix results, species distribution, and available historical records on giant ragweed invasion in Korea, we constructed seven explicit demographic scenarios (Figure 2) to model the invasion history. The first three scenarios (1–3) hypothesized a single‐source introduction from the native region, followed by divergence within Korea. Specifically, Scenario 1 assumed instantaneous divergence, whereas Scenarios 2 and 3 assumed subsequent divergence from the original single founding population. For Scenario 4, we hypothesized multiple, independent introduction events where the three specified Korean populations each independently diverged from varying genetic sources sampled in the native region. Scenario 5 tested alternative patterns of subsequent divergence following these independent introductions. The last two scenarios (Scenarios 6 and 7) incorporated multiple introductions combined with admixture events, which occurred before introduction, followed by subsequent divergence. In all scenarios, we set two key time parameters: native divergence (*t*_a_) was modeled to reflect a broad historical event, such as glacial divergence, spanning 100 < *t*_a_ < 100,000 generations/years ago. The introduction events into Korea (*t*_i_) were constrained to a relatively recent period based on occurrence records and the plant's 1‐year generation time, specifically 10 < *t*_i_ < 100 generations/years ago.

**FIGURE 2:**
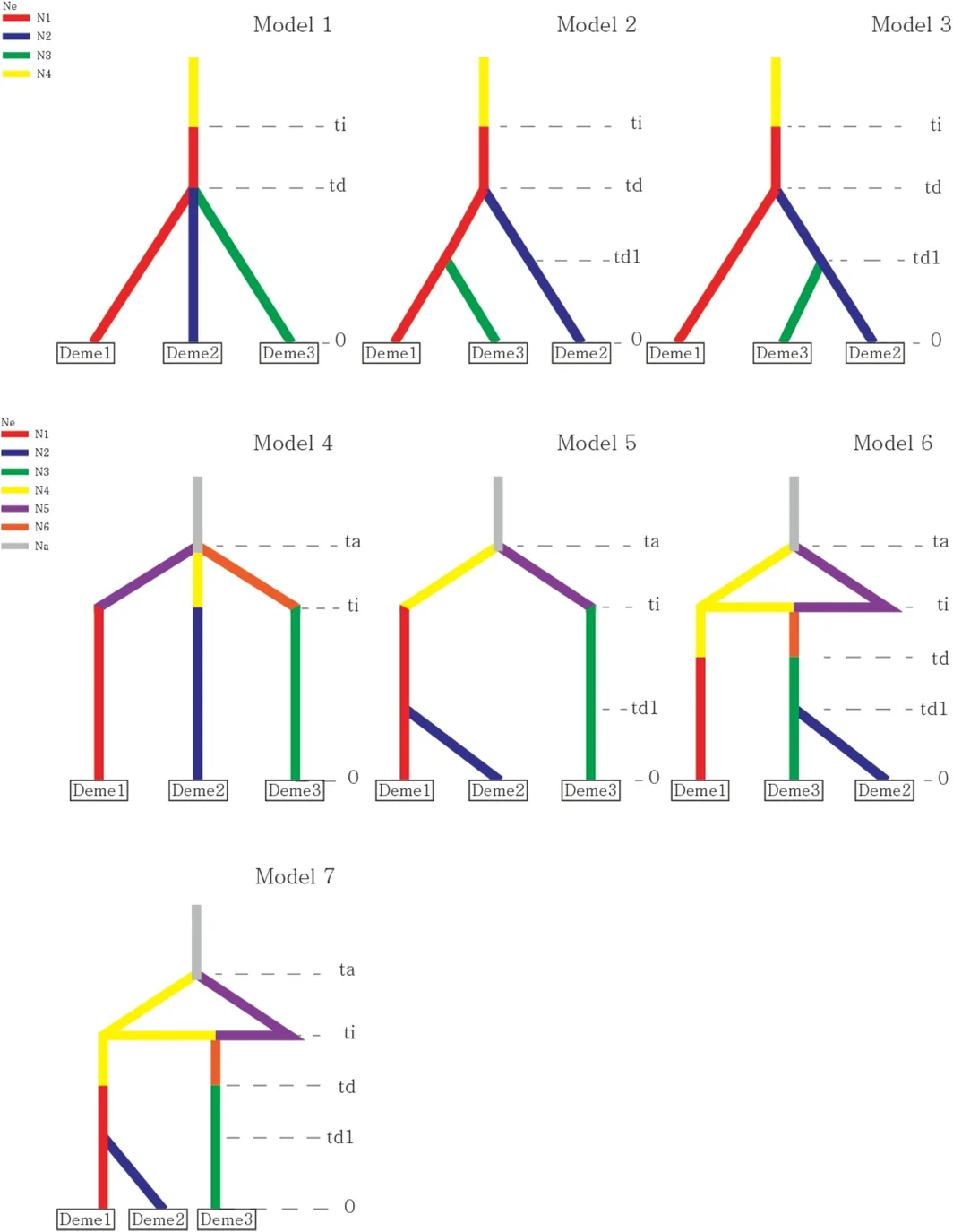
Demographic models of *Ambrosia trifida* invasion tested using Approximate Bayesian Computation (ABC). Thirty‐five Korean populations were grouped into three demes based on fastSTRUCTURE results (Table 1). Scenarios 1–3 represent single‐introduction scenarios followed by divergence among demes without hybridization. Scenarios 4 and 5 represent multiple introductions from genetically divergent source populations, with or without subsequent post‐introduction divergence. Scenarios 6 and 7 represent scenarios involving hybrid population formation following the introduction of divergent lineages, followed by divergence among demes. Parameters *t*_a_ and *t*_i_ denote divergence in the native range and the introduction event, respectively, whereas *t*_d_ and *t*_d1_ indicate post‐introduction divergence events. Parameter *r*_1_ represents the recombination rate associated with hybridization. Branch colors indicate deme‐specific effective population size (*N*_e_) parameters. Time proceeds from the oldest (top) to the most recent (bottom).

We first generated simulated data sets to validate the model and assess the accuracy of parameter estimation, visualizing the location of the observed data within the simulated prior data space using a PCA plot, as detailed in Collin et al. (2021). For the initial scenario selection phase, we simulated 4000 training data sets for each of the seven proposed scenarios, setting all parameter priors as uniform. The priors for the effective population sizes (*N*_e_) were set at 100–100,000 for sampled populations and 100–1000,000 for native and unsampled ghost populations. Divergence times (*t*ᵢ) were constrained chronologically (*t*_a_ > *t*ᵢ > *t*_d_ > *t*_d1_) within a range of 10–100 generations, whereas the ancestral divergence time at the origin (*t*_a_) was assigned a broader prior of 101–100,000 generations. The best‐fitting scenario was selected, and its posterior probability was estimated using a Random Forest (RF) algorithm implemented in DIYABC‐RF, utilizing 500 trees for the scenario choice analysis (Collin et al. 2021). Finally, the demographic parameters for the selected scenario (Scenario 5) were estimated using a regression random forest based on summary statistics with 500 trees and 100,000 simulated data.

To independently examine changes in population size, we conducted additional demographic analyses using a simulation‐based framework implemented in FASTSIMCOAL2 (Excoffier et al. 2013), separate from the ABC analyses. To minimize model complexity, three single‐population demographic models were evaluated (Figure S1): (1) a constant population size model, (2) a bottleneck model including bottleneck timing and ancestral population size, and (3) a bottleneck‐recovery model including bottleneck timing, recovery timing, ancestral population size, and bottleneck population size. A mutation rate of 7 × 10^−9^ per site per generation, estimated from *Arabidopsis thaliana* (Krasovec et al. 2018), was applied across all models. Analyses were performed separately for the three major demes identified in the population structure analyses. For each deme, folded site frequency spectra (SFS) were constructed to minimize biases arising from missing data. Composite likelihoods were estimated from 400,000 simulations using 80 expectation conditional maximization cycles, with each model run repeated 100 times and a convergence threshold of 0.001 (Excoffier et al. 2013; Excoffier and Foll 2011). Model support was evaluated using Akaike Information Criterion (AIC) values derived from the maximum‐likelihood runs. Confidence intervals for demographic parameters were obtained via parametric bootstrapping. Fifty pseudo‐replicated SFS datasets were generated from the observed SFS for each population, and FASTSIMCOAL2 analyses were repeated under the best‐supported model using identical settings. Confidence intervals were calculated from the resulting parameter distributions using R version 4.2.2 (R Core Team 2025).

## 3 Results

### 3.1 Genetic Diversity & Assignment Patterns

Each of the 558 samples yielded an average of 13.5 M raw sequence reads that passed Illumina quality filters. Following demultiplexing and trimming, we retained an average of 12 M high‐quality, 150 bp reads per sample. Approximately 0.4 million reads per sample were discarded due to missing or low‐quality restriction sites. After successive filtering steps, the final dataset comprised 3504 high‐quality SNPs from 478 individuals.

Genetic diversity varied among populations, with mean observed heterozygosity (*H*_o_) of 0.24, mean expected heterozygosity (*H*_e_) of 0.29, and mean allelic richness (*N*_a_) of 1.68 (Table 1). Genetic diversity measures differed significantly between native and introduced populations (Table 1; *p* < 0.05). Overall population differentiation was low, with a mean pairwise *F*_ST_ of 0.125 and values ranging from 0.001 (DD–DG, HS–IC, PF–PS) to 0.433 (MY–GR; Figure 3). The mean pairwise *F*_ST_ between native and introduced populations was 0.138, which was comparable to the overall level of genetic differentiation observed among all population pairs. Consistent with this pattern, isolation by distance (IBD) was not detected, as the correlation between genetic and geographic distances was weak and not statistically significant (*r* = 0.10, *p* > 0.05; Figure S2).

**FIGURE 3:**
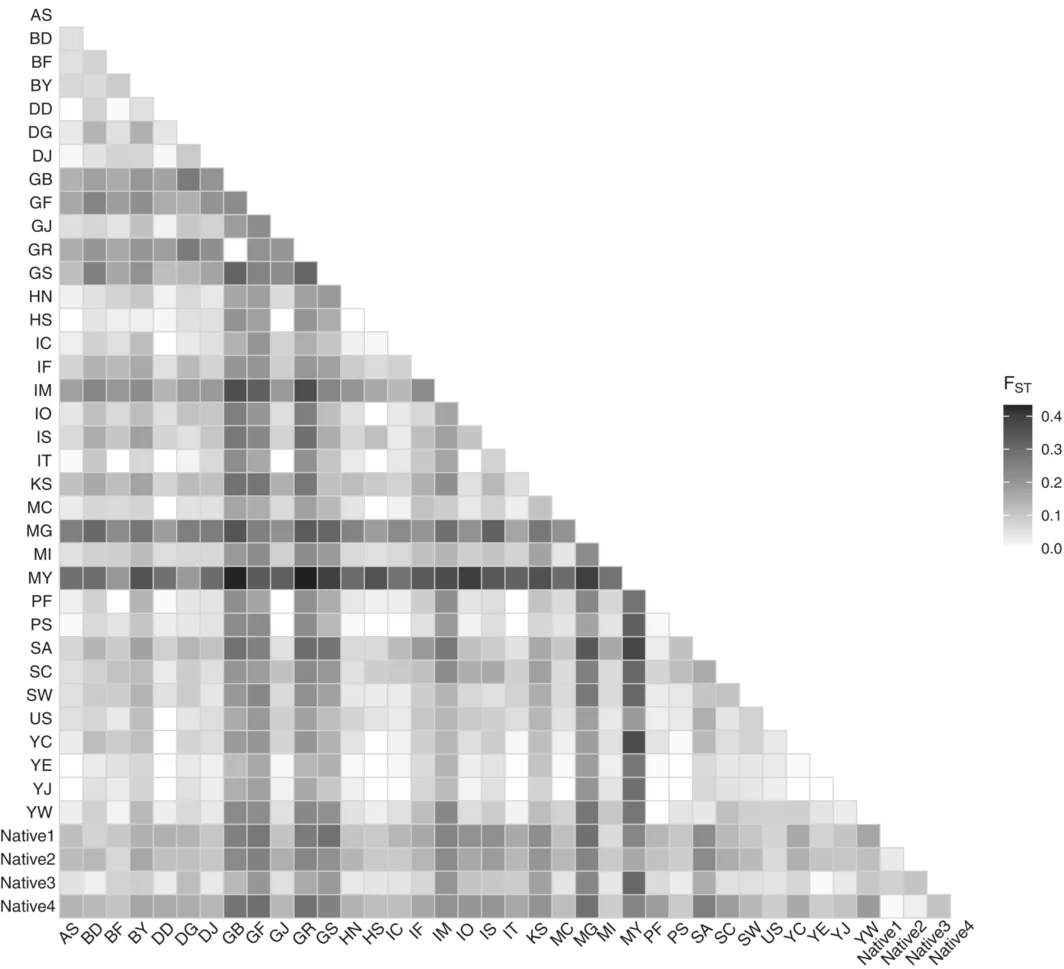
Heatmap of mean pairwise *F*_ST_ values among 39 *Ambrosia trifida* populations based on 3504 SNPs. Pairwise genetic differentiation was calculated among 35 introduced Korean populations and four native US populations. Darker shading indicates higher pairwise *F*_ST_ values, whereas lighter shading indicates lower genetic differentiation. Population acronyms are provided in Table 1.

Principal coordinate analysis (PCoA) revealed weak overall genetic structure among individuals, with the first three axes explaining 2.75% (PC1), 2.06% (PC2), and 1.74% (PC3) of the total genetic variation, respectively (Figure 4). Although the variance explained by each axis was low, the ordination broadly reflected the genetic clusters identified by the fastSTRUCTURE analysis. In both the PC1–PC2 and PC1–PC3 projections, Deme 3 was consistently separated from the remaining individuals along PC1, whereas Deme 2 was differentiated primarily along PC2 and, more distinctly, along PC3. Individuals from the native populations largely overlapped with Deme 1 and exhibited relatively little dispersion. By contrast, admixed individuals were broadly distributed across the ordination space, occupying intermediate positions between the major demes, consistent with the mixed ancestry inferred by fastSTRUCTURE.

**FIGURE 4:**
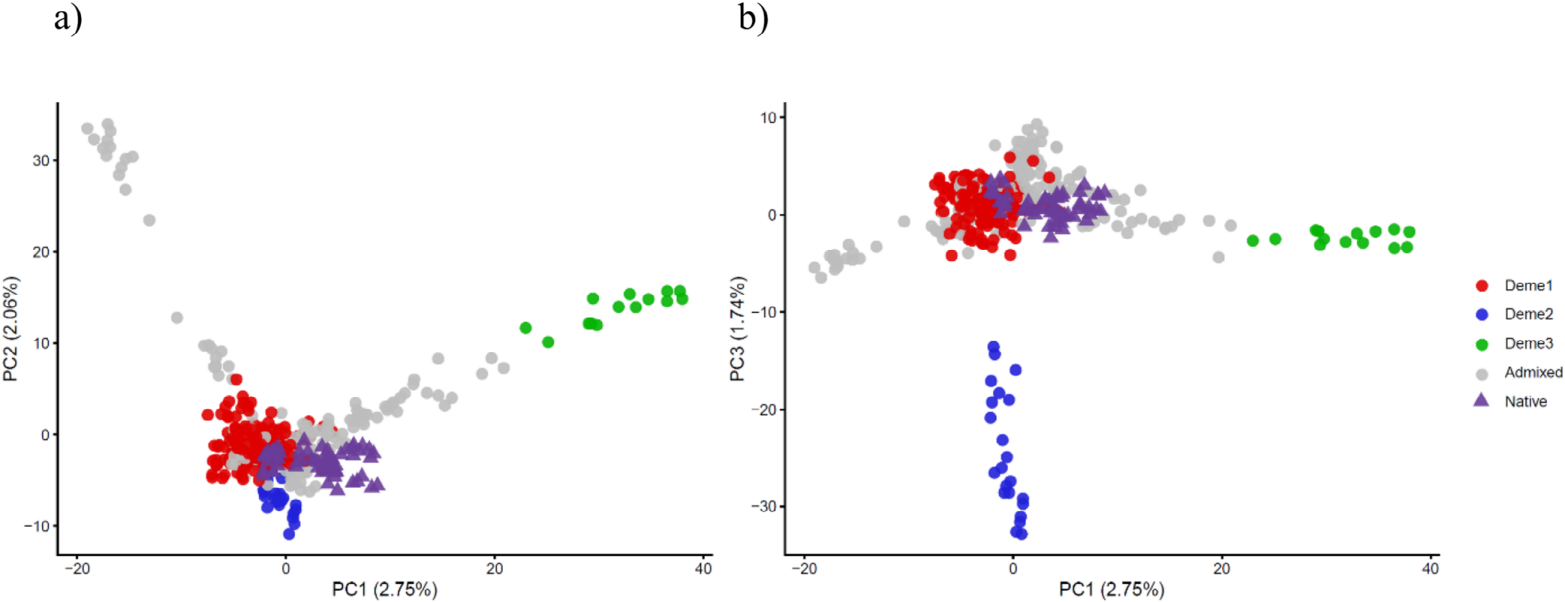
Principal coordinate analysis (PCoA) of genetic variation in 39 *Ambrosia trifida* populations, including 35 Korean and four US populations. (a) PC1 vs. PC2 plot and (b) PC1 vs. PC3 plot. Individuals are colored according to their genetic cluster assignments (see Table 1 for the deme assignments) inferred by fastSTRUCTURE.

To evaluate the robustness of the population structure analyses, we conducted two complementary sensitivity analyses. Similar to the full dataset, the downsampled PCoA analyses showed diffuse clustering with extensive overlap among individuals and no emergence of distinct genetic groups (Figure S3a). Likewise, the fastSTRUCTURE analyses recovered broadly similar ancestry patterns across replicates, with seven of the 10 replicates showing clustering patterns comparable to those observed in the full dataset at *K* = 3 (Figure S3b), indicating that the inferred population structure was robust to sample‐size imbalance. Second, we evaluated the effect of the SNP filtering strategy by applying the minor allele frequency (MAF) threshold separately to the native US samples. Although this alternative filtering retained additional native‐range polymorphisms and resulted in minor differences in genetic diversity estimates (Table S1) and individual ancestry proportions (Figure S4), the overall patterns of genetic diversity and population structure inferred from both PCoA and fastSTRUCTURE remained consistent with those obtained using the original global MAF‐filtered dataset (Figure S4). Together, these analyses indicate that the inferred patterns of genetic diversity and population structure were robust to both sample‐size imbalance and the SNP filtering strategy.

The fastSTRUCTURE analysis revealed complex population structure, with the optimal number of genetic clusters (*K*) inferred by chooseK.py ranging from *K* = 2 to 8. Because choose.K.py identified *K* = 2–8 as plausible models (Raj et al. 2014). Following the criteria described in the methods, *K* = 3 was selected for presentation because it resolved three major geographically coherent genetic lineages (Figure 1). In contrast, higher *K* values primarily subdivided these major groups without revealing additional major patterns of population subdivision. Results for alternative *K* values are provided in Figure S5. At *K* = 3, allele assignment patterns broadly corresponded to regional relationships within Korea, although notable exceptions were observed. Geographically distant population pairs such as IM and BD, and GR and GB, exhibited nearly identical genetic assignment patterns despite substantial spatial separation (Figure 1a,b). In addition, southeastern populations (MY, GB, BF, US, GJ, and KS), most of which are located in or near coastal regions, displayed distinctive clustering patterns that differed from the main inland cluster (Figure 1a). By contrast, populations in northern Korea, where *A. trifida* occurs at higher density, showed largely homogeneous assignment patterns (Figure 1a).

When the four native US populations were included, the inferred optimal *K* similarly ranged from *K* = 3 to 8, consistent with results from the Korean‐only dataset. As in the previous analysis, *K* = 3 was selected as the most informative model and showed patterns consistent with the invasion history inferred from the ABC analysis (Figure 1c; Scenario 5 in Figure 2). The four native US populations consistently clustered together, exhibiting minimal differentiation in admixture proportions (Figure 1c). Although this shared ancestry was maintained across models, varying levels of admixture were detected in all four populations across *K* = 2–10 in the supplementary bar plots (Figure S3b). Detailed fastSTRUCTURE results incorporating native populations are provided in Supporting Information.

We inferred population splits and historical migration patterns among the three major clusters identified in Korea and a cluster of the four native populations inferred from fastSTRUCTURE using TreeMix. The topology of maximum likelihood (ML) tree inferred from TreeMix was, in general, congruent with the clustering patterns of fastSTRUCTURE (Figure 5). Long branch lengths separating the major clades indicate substantial genetic drift following their divergence. Compared with the no‐migration model, which explained 95.2% of the genetic covariance, adding one migration edge greatly improved the fit of the tree to the data (99.1% of the total covariance explained; Figure S6). On the population graph, we observed one migration (migration weight = ~0.3) from Native cluster to Deme1/Deme2 cluster (Figure 5).

**FIGURE 5:**
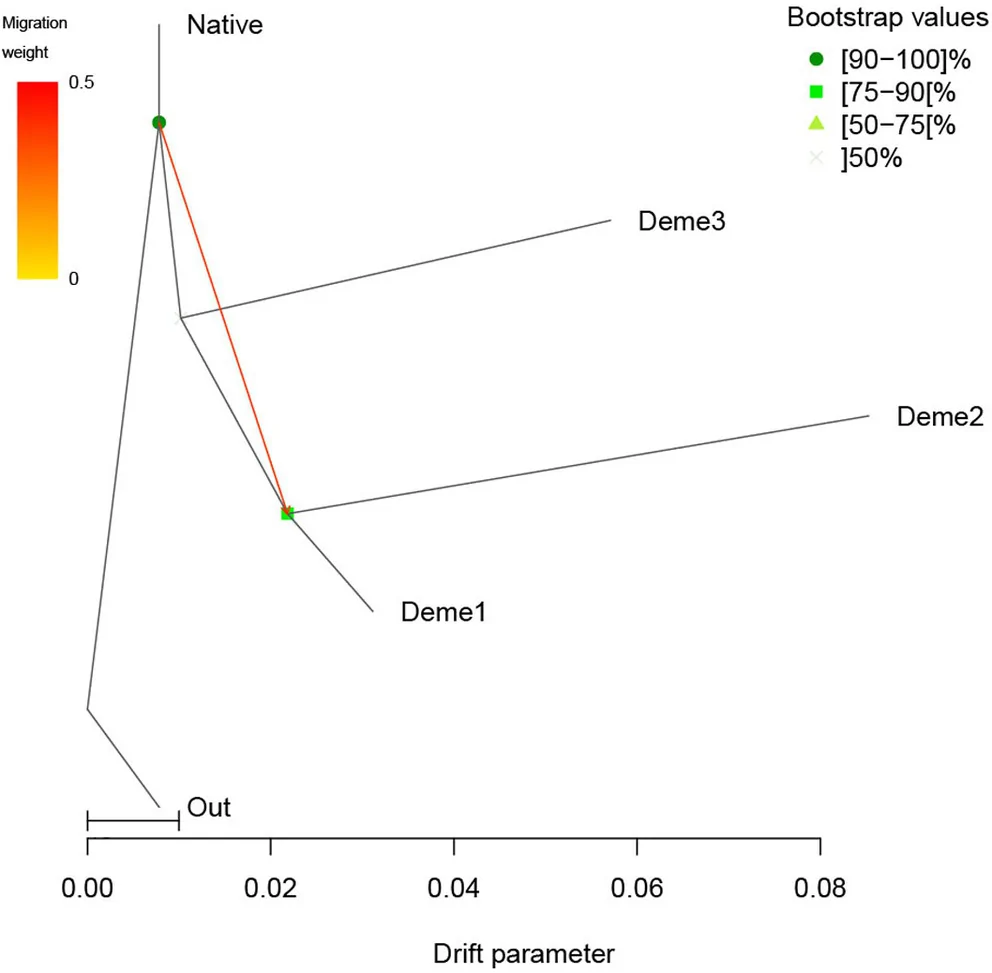
Evolutionary relationships among three Korean demes of *Ambrosia trifida* inferred using TreeMix, with native US samples included and *Ambrosia artemisiifolia* as an outgroup. The maximum‐likelihood tree was estimated allowing one migration event and explains 99.1% of the genetic covariance. Arrow direction indicates inferred gene flow, and arrow color denotes migration weight (fraction of ancestry derived from the migration edge). Node symbols represent bootstrap support.

### 3.2 Niche Analysis

The PCA‐env analysis showed extensive overlap between the climatic niches of native and introduced populations of *A. trifida* (Figure 6a). The first two principal components explained 43.2% and 24.9% of the total variation across the 17 climatic variables, respectively, and together adequately represented the available environmental space (Figure 6b). Variables contributing most strongly to PC1 were associated with colder conditions, greater temperature seasonality, and precipitation, whereas PC2 was primarily associated with higher daily temperature variation and reduced precipitation. Introduced populations largely occupied a subset of the environmental space represented in the native range, but also extended slightly toward the lower‐left region of the PCA space along the first two principal components. In contrast, a substantial portion of the native niche space was not occupied by introduced populations, indicating pronounced niche unfilling (Figure 6a and Figure S7). Niche equivalency tests based on Schoener's *D* and Warren's *I* did not reject the null hypothesis of niche equivalency (*p* = 1 for both metrics; Figure 6c). Likewise, niche similarity tests conducted in both directions were not significant (native to introduced: *D*, *p* = 0.287; introduced to native: *D*, *p* = 0.267), indicating no statistically detectable difference between native and introduced niches.

**FIGURE 6:**
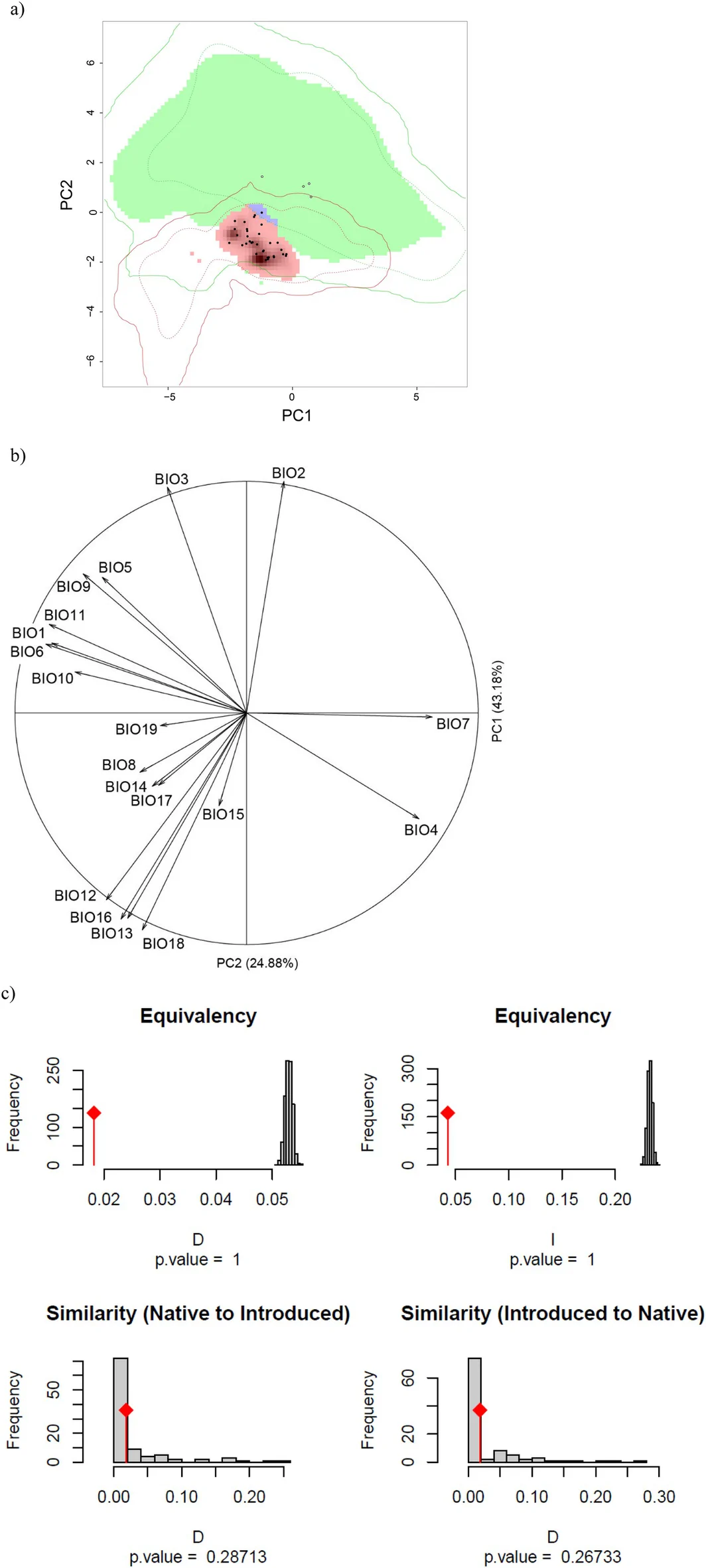
Climatic niche comparison between native and introduced ranges of *Ambrosia trifida* . (a) Niche overlap in environmental space based on PCA‐env analysis. The climatic niche occupied by native (green) and introduced (red) populations is shown as density‐smoothed occurrence distributions. The area of overlap between the two niches is indicated in blue. Solid and dashed contour lines represent 100% and 50% of the available background environmental space, respectively. Individual markers (filled circles for native and filled triangles for introduced) indicate the position of the genomically sampled populations within the environmental space. Symbols indicate the environmental coordinates of populations included in the genomic analyses, with open symbols representing the four native US populations and filled symbols representing the 35 introduced Korean populations. (b) Correlation circle showing the contribution and direction of the 19 bioclimatic variables (BIO1–BIO19) with respect to the first two principal components, which together define the environmental space used for subsequent niche analyses. (c) Results of niche equivalency and similarity tests based on Schoener's *D* and Warren's *I*. Grey histograms represent null distributions generated by randomization, and red diamonds indicate the observed overlap values between native and introduced niches. Reported *p*‐values correspond to tests of niche equivalency and bidirectional niche similarity.

### 3.3 Inferring Invasion History

Among the seven demographic models evaluated using ABC (Figure 2), Scenario 5 received the strongest support. Random forest model selection favored this scenario with 430 of 500 classification votes and a posterior probability of 0.85 (Table S2). Scenario 5 assumes multiple introductions from genetically divergent source populations, followed by sequential divergence among Korean demes. In linear discriminant analysis, the observed dataset was clearly separated from alternative models along the first two axes (Figure S8). However, global and local out of bag error rates of 0.30 and 0.10 indicate moderate discriminatory power among competing scenarios (Table S2). Summary statistics contributing most strongly to model selection included pairwise *F*_ST_ and related metrics, linear discriminant axes, and heterozygosity‐based measures (Figure S9).

Parameter estimation under the selected scenario was conducted using partial least squares (PLS) axes to improve predictive accuracy (Collin et al. 2021). This approach resulted in relatively low normalized mean absolute errors (NMAE) at both global and local scales (Table 2). The mean estimated time of introduction (*t*_i_) was 65 generations, followed by subsequent divergence among demes at a mean time (*t*_d_) of 45 generations (Table 2). Effective population size estimates for the three inferred demes were approximately 90,000, 29,000, and 500 individuals, respectively (Table 2). Overall, parameter estimates were characterized by narrow 90% credibility intervals and low associated error rates, indicating robust inference under the selected model (Table 2).

**TABLE 2:** Posterior estimates of demographic parameters inferred from Approximate Bayesian Computation (ABC) under the best‐supported demographic scenario (Scenario 5) for *Ambrosia trifida* invasion.

Parameter		Expectation	Median	Quantile 0.05	Quantile 0.95	Global NMAE	Local NMAE
Effective population size	*N*_a_	591,852	597,072	442,793	885,214	0.1922	0.1169
	*N*_1_	89,713.3	93,321	58,821.4	98,213.9	0.1991	0.1122
	*N*_2_	28,855.1	27,521	25,841.8	48,232.2	0.1932	0.0677
	*N*_3_	497	560	319.8	874.5	0.1412	0.0551
	*N*_4_	448,297	465,289	400,886.5	493,675	0.1865	0.1125
	*N*_5_	224,587	230,501	103,852.6	401,288.1	0.2266	0.1872
Time of divergence	*t*_a_	822	884	768	996.4	0.3009	0.2134
	*t*_i_	64.8	67	55.2	77	0.1994	0.1299
	*t*_d1_	14.7	14	10	16.6	0.1754	0.1122
Recombination rate	*r*	0.627	0.648	0.597	0.662	0.2362	0.1246

*Note:* For each parameter, posterior expectation, median, the 5% and 95% quantiles of the posterior distribution and normalized mean absolute errors are reported. Model parameters included effective population sizes for the ancestral population and derived demes (*N*_a_, *N*_1_–*N*_5_; see Table 1 for deme assignments), divergence and demographic event times (*t*_a_, *t*_i_, *t*_d_, and *t*_d1_; expressed in generations), and the recombination rate (*r*). Effective population sizes are expressed as numbers of diploid individuals, and quantiles represent 90% credible intervals.

Inference from the selected demographic model suggests that the Korean populations of *A. trifida* originated from at least two genetically divergent source populations, including the sampled North Central US populations, which most likely represent one of the principal source lineages contributing to the Korean invasion (Figure 1c). The introduction was dated to approximately 65 generations ago, assuming one generation per year (Table 2). Following the initial introduction of the major deme, divergence among founding populations occurred approximately 20 generations later, giving rise to a secondary deme. Among the three demographic models evaluated with FASTSIMCOAL2, Model 3 consistently provided the best fit to the data, exhibiting the lowest AIC values across all three demes (Table S3). Based on these reconstructions, Demes 1 and 3 experienced severe population bottlenecks immediately after initial introduction, with substantial population expansion beginning approximately 50 generations ago (Table 3; Figure S1). Deme 2 also showed evidence of a recent bottleneck around 15 generations ago, followed by population expansion beginning approximately 10 generations ago (Table 3; Figure S1).

**TABLE 3:** Parameter estimates of the selected demographic model (Model 3, see Figure S1 for the model details) inferred from coalescent simulations in FASTSIMCOAL2 for three *Ambrosia trifida* demes identified by fastSTRUCTURE during the invasion process (see Table 1 for the deme assignments).

Deme	*N*_CON_	*N*_ANC_	*N*_BOT_	*T*_BOT1_	*T*_BOT2_
1	95,733 [95,022, 97,885]	310,463 [300,682, 352,311]	669 [524, 993]	67 [60, 72]	57 [55, 62]
2	30,925 [25,098, 32,562]	102,367 [98,245, 153,782]	188 [172, 322]	15 [12, 15]	12 [8, 13]
3	423 [389, 788]	7885 [5228, 8021]	28 [22, 39]	68 [62, 74]	58 [57, 77]

*Note:* All time parameters are estimated backward from the present. Values are shown with lower and upper confidence intervals in brackets.

Abbreviations: *N*_ANC_, the effective size prior to the bottleneck; *N*_BOT_, effective population size during the bottleneck; *T*_BOT1_, end of population decline; *T*_BOT2_, onset of population decline.

## 4 Discussion

Reconstructing invasion history is critical for understanding the processes shaping genetic variation in introduced populations, yet such histories remain poorly resolved for many unintentionally introduced species. By integrating population genomic data with climatic niche comparisons, we show that the rapid spread of *A. trifida* in Korea reflects a complex invasion history involving multiple introductions followed by range expansion. Despite evidence of recent demographic bottlenecks, introduced populations retained relatively high genetic diversity, consistent with admixture among genetically divergent source populations. Patterns of genetic structure across native and introduced ranges, together with demographic reconstructions, support repeated introductions and subsequent human‐mediated dispersal within Korea. Collectively, these results clarify how introduction history and post‐introduction processes have shaped contemporary genetic structure in *A. trifida* .

### 4.1 Genetic Diversity & Assignment Patterns

Invasive species commonly experience reductions in genetic diversity due to founder effects and demographic bottlenecks during establishment (Dlugosch and Parker 2008; Dlugosch et al. 2015; Hodgins et al. 2025). In their synthesis of 80 invasive taxa, Dlugosch and Parker (2008) reported significant losses of allelic richness in more than half of the cases examined. Contrary to this general expectation, we did not detect a reduction in genetic diversity within the introduced range of giant ragweed in Korea. Instead, genomic analyses revealed slightly but significantly higher levels of genetic diversity in Korean populations compared to native populations, as measured by both expected heterozygosity and allelic richness (*t*‐tests, *p* < 0.05). This unexpected genetic variation in introduced populations strongly mirrors patterns documented in the closely related common ragweed ( *A. artemisiifolia* ) across Europe, Australia, and China (Genton et al. 2005; Chun et al. 2009; Van Boheemen et al. 2017; Li et al. 2019). In *A. artemisiifolia* , multiple independent introductions from genetically differentiated native clades have been shown to quickly counteract local founder effects, elevating genetic diversity through inter‐population admixture (Chun et al. 2009; Li et al. 2019). Because both *Ambrosia* species are highly outcrossing, wind‐pollinated annuals that produce long‐lived, highly viable seeds, they are biologically primed to maintain and rapidly redistribute genetic variation once initial bottlenecks are bypassed.

Indeed, maintenance or even enhancement of genetic variation during invasion is typically driven by either repeated introductions and subsequent admixture, or large initial propagule pressure (Lynch and Conery 2000; Novak and Mack 2005; Dlugosch and Parker 2008; Simberloff 2009; Bock et al. 2015; Dlugosch et al. 2015; Kołodziejczyk et al. 2025). Our population structure and demographic analyses support the former mechanism as a primary driver of genetic diversity in Korean giant ragweed, with substantial admixture originating from native source populations and reinforced by multiple human‐mediated introductions. Nevertheless, the comparison of genetic diversity between the introduced and native ranges should be interpreted in light of the limited and geographically uneven sampling of the native range. In particular, all four sampled native populations were genetically associated with Deme 1, and therefore may not capture the full extent of genetic variation present across the broad native distribution of *A. trifida* . This limitation could influence estimates of mean genetic diversity and the ability to identify specific native sources for the other Korean demes. However, our sensitivity analyses addressing the imbalance in native and introduced sampling produced highly consistent patterns of genetic diversity and population structure, indicating that the major conclusions were not strongly affected by the unequal sample sizes. Thus, although broader sampling of the native range would improve resolution of source attribution, the observed genetic patterns and evidence for multiple introductions are unlikely to be explained solely by the sampling imbalance.

Human activities are widely recognized as key drivers of invasive species dispersal because they establish transport pathways and repeatedly move propagules across large distances, thereby reshaping spatial genetic structure (Bertelsmeier and Keller 2018; Bertelsmeier et al. 2018; Mairal et al. 2021). Under such conditions, human‐mediated dispersal is expected to weaken spatial genetic differentiation by facilitating gene flow among geographically distant populations (Wilson et al. 2009). Consistent with this expectation, our genomic analyses reveal generally weak spatial genetic structure across much of Korea, reflected by the widespread dominance of a single major genetic cluster rather than strong population‐specific differentiation. This pattern is most pronounced in populations surrounding the Seoul metropolitan area, where most populations are largely assigned to the same deme, suggesting extensive secondary spread driven by anthropogenic transport networks.

In contrast, populations in the southeastern and eastern regions retain more distinct genetic signatures. These patterns are consistent with ABC‐based demographic inference indicating multiple introductions from at least two genetically divergent source populations. One source contributed predominantly to the major deme that now dominates much of Korea, whereas another gave rise to a secondary deme that subsequently diverged from the first, with an additional deme originating from a separate source. The persistence of distinct genetic signatures in southeastern and eastern populations indicates that genetic contributions from different introduction sources have remained regionally structured rather than becoming fully homogenized through subsequent spread. Together, these results underscore the combined influence of multiple introductions and human‐mediated dispersal in shaping the genetic structure of giant ragweed during its invasion of Korea.

### 4.2 Niche Dynamics and Non‐Equilibrium Distribution

Predicting the potential geographic extent of invasive species is critical for robust risk assessment, yet distributions frequently remain far from the migration‐drift equilibrium during early to intermediate stages of invasion (Václavík and Meentemeyer 2012; Putra et al. 2024). When species distributions are at equilibrium, populations occupy all suitable environments, a condition more commonly assumed for native ranges or long‐established introductions (Guisan and Thuiller 2005). At such equilibrium stages, a significant IBD pattern is expected, producing a positive correlation between genetic and geographic distance (Kimura 1964; Cox and Durrett 2002; Briscoe Runquist and Moeller 2024).

In our genomic analysis, the absence of significant IBD patterns suggests that Korean *A. trifida* populations likely have not yet reached a state of equilibrium. This finding is further supported by our climatic niche analyses, which indicate that the species is still actively expanding its environmental occupancy. Our PCA‐env results (Figure 6a) reveal substantial niche unfilling toward warmer climates characterized by greater temperature variability, whereas simultaneously detecting niche expansion into wetter climatic conditions in the introduced range (Figure S4). These patterns are consistent with a shift in the realized climatic niche during the invasion process, indicating that the current distribution of *A. trifida* in Korea may not yet reflect its full climatic potential. Similar niche shifts have been reported in other invasive plant species, including the closely related *A. artemisiifolia* (Chapman et al. 2017), and climatic niche dynamics have been recognized as an important component of biological invasions (Atwater et al. 2017).

When interpreting these species‐level macro‐ecological dynamics, the underlying genetic structure of the founding populations remains an important consideration. For instance, in the closely related *A. artemisiifolia* , genomically‐informed niche modeling demonstrates that distinct genetic clusters carry variable, pre‐adapted climatic tolerances that dictate localized potential distributions and invasion success (Putra et al. 2024). Although our current study focuses on the aggregate niche shift of *A. trifida* in Korea relative to the native US range, the rapid demographic splitting inferred by our ABC models raises the possibility that genetically divergent lineages may contribute differently to future range expansion, although testing this hypothesis will require lineage‐specific ecological niche analyses. If the distinct genetic demes introduced to Korea harbor unique environmental tolerances, further range expansion and subsequent admixture among these demes could continue to drive the observed niche expansion.

The PCA‐env patterns should nevertheless be interpreted in light of the niche equivalency and similarity tests. Although the observed overlap between the native and introduced niches was low (*D* < 0.1, *I* < 0.1), the niche equivalency tests were not statistically significant (*p* = 1.0), and the similarity tests were also non‐significant (*p* > 0.05). Thus, the observed differences in climatic niche occupancy cannot be interpreted as evidence of a statistically supported niche shift. Rather, the PCA‐env results indicate differences in the portions of environmental space occupied by the native and introduced ranges, whereas the statistical tests indicate that these differences are not sufficient to reject niche equivalency or distinguish the observed overlap from the corresponding null expectations.

### 4.3 Inferring Invasion History

Compared with common ragweed ( *A. artemisiifolia* ), giant ragweed ( *A. trifida* ) became problematic in Europe and East Asia more recently, beginning in the late nineteenth century, yet it has spread rapidly and attained invasive status across much of its introduced range (Lee and Oh 1974; Qin et al. 2014; Kim and Kil 2016; EPPO 2020). In Korea, the invasion history of *A. trifida* spans approximately five decades, with expansion accelerating markedly over the past 25 years and leading to increasing ecological and management concerns (Kim and Kil 2016; Cho et al. 2022). Despite this rapid spread, the origin, timing, and dispersal pathways of *A. trifida* in Korea have remained poorly resolved due to the largely unintentional nature of its introduction and the scarcity of reliable historical records (Kil et al. 2004; Kim and Kil 2016). Our ABC analyses provide new insights into the invasion history of *A. trifida* in Korea by identifying likely source populations, introduction timing, and subsequent expansion pathways. The choice to model these introductions as complex, multi‐source events with distinct bottleneck phases is heavily supported by demographic modeling in *A. artemisiifolia* , where ABC analyses have repeatedly demonstrated that multi‐source scenarios yield significantly higher model support than simple single‐origin models (Van Boheemen et al. 2017; Battlay et al. 2025).

The best‐supported demographic scenario indicates a recent introduction event giving rise to the primary Korean deme (Deme 1) approximately 65 years ago, followed by divergence among demes within the last ~15 years. These estimates broadly align with available herbarium and historical records, despite their incompleteness. Importantly, the ABC results support multiple independent introductions from genetically divergent source populations, including a sampled source from the North Central US, a recognized genetic diversity hotspot for *A. trifida* , as well as at least one additional unsampled source.

Historical context further supports these inferences. Although the timing and pathway of *A. trifida* introduction to Korea remain uncertain, post Korean War introduction via US military base camps has been suggested. Notably, three of the four major US Army bases historically present in Korea are located near the Seoul metropolitan region, where Deme 1 is now widespread, whereas the remaining base is situated near Daegu, corresponding to the more localized distributions of Demes 2 and 3. Together with our genomic evidence, these observations suggest that the dominant Korean deme was likely founded by introductions from North Central US populations, whereas Deme 3 may reflect a separate introduction from an unsampled source associated with the Daegu region. Across invasive plant systems, repeated introductions are commonly associated with elevated effective population sizes and increased genetic variation in introduced ranges, particularly when source populations are geographically structured. Consistent with this broader pattern, the Korean invasion of *A. trifida* appears to reflect multiple introduction events followed by admixture and rapid expansion, processes that have collectively shaped contemporary genetic structure across the invaded range.

## 5 Conclusion

Our analyses indicate that the invasion of *A. trifida* in Korea has been driven primarily by human‐mediated processes rather than by major ecological or climatic niche shifts. Population genomic and demographic reconstructions support a recent introduction involving multiple, genetically divergent sources, followed by rapid secondary spread. Despite founder events, high effective population sizes and extensive admixture suggest that demographic constraints were quickly overcome during establishment. Climatic niche comparisons show that the current Korean distribution largely overlaps with the native climatic space, implying that invasion success has been shaped mainly by introduction history and anthropogenic dispersal. From a management perspective, these results highlight the importance of limiting further introductions and secondary dispersal, particularly into climatically suitable but currently unoccupied regions, to reduce the long‐term spread and persistence of giant ragweed in Korea.

## Funding

This work was supported by Korea National Arboretum (KNA1‐2‐53, 26‐5) and Chosun University, 2024.

## Ethics Statement

All plant materials were collected in accordance with relevant institutional, national, and international guidelines and legislation. Field sampling was conducted with appropriate permits where required, and no protected species were collected.

## Conflicts of Interest

The authors declare no conflicts of interest.

## Supporting information


**Table S1:** Comparison of genetic diversity estimates obtained using the original global minor allele frequency (MAF) filtering strategy and the alternative filtering strategy in which the MAF threshold was applied separately to the native US samples. Estimates of genetic diversity were highly consistent between the two filtering approaches, indicating that the principal conclusions of the study are robust to the SNP filtering strategy.
**Table S2:** Model selection results from Approximate Bayesian Computation (ABC) analyses for *Ambrosia trifida* . Seven alternative demographic scenarios were evaluated, and support for each scenario is shown as the proportion of votes obtained from the ABC model selection procedure. The posterior probability, global error rate, and local error rate are reported for the best‐supported scenario, indicating the confidence and reliability of scenario discrimination.
**Table S3:** Akaike Information Criterion (AIC) values for three demographic models fitted using FASTSIMCOAL2 for the three *Ambrosia trifida* demes identified by fastSTRUCTURE, used for demographic model comparison.
**Figure S1:** Historical changes in effective population size (*N*_e_) of three *Ambrosia trifida* demes identified by fastSTRUCTURE and inferred using simulation‐based demographic analyses implemented in FASTSIMCOAL2. Model parameters include *N*_ANC_ (ancestral effective population size), *N*_CON_ (contemporary effective population size), *N*_BOT_ (Bottlenecked effective population size), *T*_BOT1_ (time of bottleneck initiation), and *T*_BOT2_ (time of bottleneck termination).
**Figure S2:** Relationship between geographic distance and pairwise genetic differentiation (*F*_ST_) among 35 Korean populations of *Ambrosia trifida* . Isolation by distance was evaluated using a Mantel test based on linearized *F*_ST_ values and Euclidean geographic distances, and no significant correlation was detected (*r* = 0.10, *p* > 0.05).
**Figure S3:** Sensitivity analyses using downsampled introduced samples. To evaluate the potential effect of unequal sample sizes between native (*n* = 59) and introduced (*n* = 419) samples, 10 independent downsampling replicates were generated by randomly selecting 59 Korean individuals to match the native sample size. (a) Three representative principal coordinate analysis (PCoA) plots from the 10 downsampling replicates. Similar to the full dataset, the downsampled datasets showed diffuse clustering and substantial overlap among individuals. (b) fastSTRUCTURE bar plots (*K* = 3) for all 10 downsampling replicates. Most replicates recovered genetic assignment patterns comparable to those obtained from the full dataset, indicating that the inferred population structure is robust to sample‐size imbalance.
**Figure S4:** Sensitivity analyses using an alternative minor allele frequency (MAF) filtering strategy. To evaluate the potential effect of global MAF filtering on the representation of native‐range variation, the MAF threshold was applied separately to the native US samples, and the resulting dataset was compared with the original globally filtered dataset. (a) Principal coordinate analysis (PCoA) of the alternatively filtered dataset. Similar to the original dataset, the ordination showed diffuse clustering and substantial overlap among individuals. (b) fastSTRUCTURE bar plots based on the alternatively filtered dataset. The overall genetic assignment patterns were comparable to those obtained using the original global MAF filtering strategy, indicating that the inferred population structure was robust to the SNP filtering approach.
**Figure S5:** Bayesian clustering analysis of *Ambrosia trifida* inferred using fastSTRUCTURE for *K* = 2–10. (a) Ancestry proportions for 35 Korean (KR) populations. (b) Ancestry proportions for the complete dataset including both 35 Korean and four native US populations. Each vertical bar represents an individual, with colors indicating proportional membership in inferred genetic clusters. Populations are separated by dashed white lines and labeled according to the population codes in Table 1.
**Figure S6:** Model selection for TreeMix analysis based on the number of migration edges (*m*). The plot shows changes in log‐likelihood as a function of *m* (0–5). Change points were evaluated using piecewise linear, bent‐cable, and simple exponential models to identify the optimal number of migration events. Allowing a single migration edge (*m* = 1) resulted in the largest improvement in model fit, after which log‐likelihood values plateaued.
**Figure S7:** Density distributions of 19 bioclimatic variables in native (green) and introduced (red) environmental spaces for *Ambrosia trifida* . Shaded areas represent occurrence densities, whereas solid and dashed lines indicate the available environmental background in each range. Blue arrows denote the direction and magnitude of niche shifts between native and introduced ranges.
**Figure S8:** Linear discriminant analysis (LDA) plot projecting simulated training datasets and the observed dataset onto the first two LDA axes based on summary statistics for the selected demographic scenario.
**Figure S9:** Relative contribution of the 30 most informative summary statistics to the random forest used for model selection among the nine demographic scenarios.

## Data Availability

The data that support the findings of this study are openly available in GenBank at https://www.ncbi.nlm.nih.gov/genbank/, reference number PRJNA12222347.

## References

[eva70333-bib-0001] Abul, H. A. , and F. A. Bazzaz . 1979. “The Biology of *Ambrosia trifida* L. I. Influence of Species Removal on the Organization of the Plant Community.” New Phytologist 83: 813–816.

[eva70333-bib-0002] Allard, H. A. 1943. “The North American Ragweeds and Their Occurrence in Other Parts of the World.” Science 98: 292–294. 10.1126/science.98.2544.292 17776054

[eva70333-bib-0108] Atwater, D. Z. , C. Ervine , and J. N. Barney . 2017. “Climatic Niche Shifts are Common in Introduced Plants.” Nature Ecology & Evolution 2, no. 1: 34–43. 10.1038/s41559-017-0396-z. 29203919

[eva70333-bib-0004] Bassett, I. J. , and C. W. Crompton . 1982. “The Biology of Canadian Weeds. 55. *Ambrosia trifida* L.” Canadian Journal of Plant Science 62: 1003–1010.

[eva70333-bib-0098] Battlay, P. , S. Craig , A. R. Putra , et al. 2025. “Rapid Parallel Adaptation in Distinct Invasions of *Ambrosia Artemisiifolia* is Driven by Large‐Effect Structural Variants.” Molecular Biology and Evolution 42, no. 1: msae270. 10.1093/molbev/msae270. PMC11733498 39812008

[eva70333-bib-0005] Bayona‐Vásquez, N. J. , T. C. Glenn , T. J. Kieran , et al. 2019. “Adapterama III: Quadruple‐Indexed, Double/Triple‐Enzyme RADseq Libraries (2RAD/3RAD).” PeerJ 7: e7724. 10.7717/peerj.7724 PMC6791345 31616583

[eva70333-bib-0006] Bazzaz, F. A. , and R. W. Carlson . 1979. “Photosynthetic Contribution of Flowers and Seeds to Reproductive Effort of an Annual Colonizer.” New Phytologist 82: 223–232.

[eva70333-bib-0007] Bertelsmeier, C. , and L. Keller . 2018. “Bridgehead Effects and Role of Adaptive Evolution in Invasive Populations.” Trends in Ecology & Evolution 33: 527–534. 10.1016/j.tree.2018.04.014 29764688

[eva70333-bib-0008] Bertelsmeier, C. , S. Ollier , A. M. Liebhold , E. G. Brockerhoff , D. Ward , and L. Keller . 2018. “Recurrent Bridgehead Effects Accelerate Global Alien Ant Spread.” Proceedings of the National Academy of Sciences of the United States of America 115: 5486–5491. 10.1073/pnas.1801990115 PMC6003495 29735696

[eva70333-bib-0009] Blackburn, T. M. , J. L. Lockwood , and P. Cassey . 2015. “The Influence of Numbers on Invasion Success.” Molecular Ecology 24: 1942–1953. 10.1111/mec.13075 25641210

[eva70333-bib-0010] Bock, D. G. , C. Caseys , R. D. Cousens , et al. 2015. “What We Still Don't Know About Invasion Genetics.” Molecular Ecology 24: 2277–2297. 10.1111/mec.13032 25474505

[eva70333-bib-0011] Briscoe Runquist, R. , and D. A. Moeller . 2024. “Isolation by Environment and Its Consequences for Range Shifts With Global Change: Landscape Genomics of the Invasive Plant Common Tansy.” Molecular Ecology 33: 1–16. 10.1111/mec.17462 38993027

[eva70333-bib-0012] Broennimann, O. , M. C. Fitzpatrick , P. B. Pearman , et al. 2012. “Measuring Ecological Niche Overlap From Occurrence and Spatial Environmental Data.” Global Ecology and Biogeography 21: 481–497.

[eva70333-bib-0107] Chapman, D. S. , R. Scalone , E. Štefanić , and J. M. Bullock . 2017. “Mechanistic Species Distribution Modeling Reveals a Niche Shift During Invasion.” Ecology 98, no. 6: 1671–1680. 10.1002/ecy.1835. 28369815

[eva70333-bib-0013] Chauvel, B. , G. Fried , S. Follak , et al. 2021. “Monographs on Invasive Plants in Europe No. 5: *Ambrosia trifida* L.” Botany Letters 168: 167–190.

[eva70333-bib-0014] Chauvel, B. , A. Rodriguez , C. Moreau , Q. Martinez , R. Bilon , and G. Fried . 2015. “Spread of *Ambrosia trifida* L. in France: Historical and Ecological Knowledge for the Eradication of the Species.” Journal of Botany 71: 2538.

[eva70333-bib-0111] Cho, K. H. , J.‐S. Park , J. H. Kim , Y. S. Kwon , and D.‐H. Lee . 2022. “Modeling the Distribution of Invasive Species (*Ambrosia* spp.) Using Regression Kriging and Maxent.” Frontiers in Ecology and Evolution 10: 1036816. 10.3389/fevo.2022.1036816.

[eva70333-bib-0094] Chun, Y. J. , B. Fumanal , B. Laitung , and F. Bretagnolle . 2009. “Gene Flow and Population Admixture as the Primary Post‐Invasion Processes in Common Ragweed (*Ambrosia artemisiifolia*) Populations in France.” New Phytologist 185, no. 4: 1100–1107. 10.1111/j.1469-8137.2009.03129.x. 20028474

[eva70333-bib-0015] Collin, F. D. , G. Durif , L. Raynal , et al. 2021. “Extending Approximate Bayesian Computation With Supervised Machine Learning to Infer Demographic History Using DIYABC Random Forest.” Molecular Ecology Resources 21: 2598–2613. 10.1111/1755-0998.13413 PMC8596733 33950563

[eva70333-bib-0016] Cornuet, J. M. , V. Ravigné , and A. Estoup . 2010. “Inference on Population History and Model Checking Using DNA Sequence and Microsatellite Data With DIYABC.” BMC Bioinformatics 11: 401. 10.1186/1471-2105-11-401 PMC2919520 20667077

[eva70333-bib-0017] Cox, J. T. , and R. Durrett . 2002. “The Stepping Stone Model: New Formulas Expose Old Myths.” Annals of Applied Probability 12: 1348–1377.

[eva70333-bib-0018] Cristescu, M. E. 2015. “Genetic Reconstructions of Invasion History.” Molecular Ecology 24: 2212–2225. 10.1111/mec.13117 25703061

[eva70333-bib-0019] Davis, W. E. 1930. “Primary Dormancy, After‐Ripening and the Development of Secondary Dormancy in Embryos of *Ambrosia trifida* .” American Journal of Botany 17: 58–76.

[eva70333-bib-0020] Di Cola, V. , O. Broennimann , B. Petitpierre , et al. 2017. “Ecospat: An R Package to Support Spatial Analyses and Modelling of Species Niches and Distributions.” Ecography 40: 774–787.

[eva70333-bib-0021] Diagne, C. , B. Leroy , A. C. Vaissière , et al. 2021. “High and Rising Economic Costs of Biological Invasions Worldwide.” Nature 592: 571–576. 10.1038/s41586-021-03405-6 33790468

[eva70333-bib-0022] Dlugosch, K. M. , S. R. Anderson , J. Braasch , F. A. Cang , and H. D. Gillette . 2015. “The Devil Is in the Details: Genetic Variation in Introduced Populations and Its Contributions to Invasion.” Molecular Ecology 24: 2095–2111. 10.1111/mec.13183 25846825

[eva70333-bib-0023] Dlugosch, K. M. , and I. M. Parker . 2008. “Founding Events in Species Invasions: Genetic Variation, Adaptive Evolution and the Role of Multiple Introductions.” Molecular Ecology 17: 431–449. 10.1111/j.1365-294X.2007.03538.x 17908213

[eva70333-bib-0025] EPPO . 2020. “ *Ambrosia trifida* L.” EPPO Bulletin 50: 243–248.

[eva70333-bib-0026] Estoup, A. , and T. Guillemaud . 2010. “Reconstructing Routes of Invasion Using Genetic Data: Why, How and So What?” Molecular Ecology 19: 4113–4130. 10.1111/j.1365-294X.2010.04773.x 20723048

[eva70333-bib-0027] Estoup, A. , V. Ravigné , R. Hufbauer , R. Vitalis , M. Gautier , and B. Facon . 2016. “Is There a Genetic Paradox of Biological Invasion?” Annual Review of Ecology, Evolution, and Systematics 47: 51–72.

[eva70333-bib-0103] Excoffier, L. , I. Dupanloup , E. Huerta‐Sánchez , V. C. Sousa , and M. Foll . 2013. “Robust Demographic Inference From Genomic and SNP Data.” PLoS Genetics 9, no. 10: e1003905. 10.1371/journal.pgen.1003905. PMC3812088 24204310

[eva70333-bib-0104] Excoffier, L. , and M. Foll . 2011. “Fastsimcoal: A Continuous‐Time Coalescent Simulator of Genomic Diversity Under Arbitrarily Complex Evolutionary Scenarios.” Bioinformatics 27, no. 9: 1332–1334. 10.1093/bioinformatics/btr124. 21398675

[eva70333-bib-0028] Excoffier, L. , and H. E. L. Lischer . 2010. “Arlequin Suite ver. 3.5: A New Series of Programs to Perform Population Genetics Analyses.” Molecular Ecology Resources 10: 564–567. 10.1111/j.1755-0998.2010.02847.x 21565059

[eva70333-bib-0029] Fick, S. E. , and R. J. Hijmans . 2017. “WorldClim 2: New 1‐km Spatial Resolution Climate Surfaces for Global Land Areas.” International Journal of Climatology 37: 4302–4315.

[eva70333-bib-0030] Fitak, R. R. 2021. “OptM: Estimating the Optimal Number of Migration Edges on Population Trees Using TreeMix.” Biology Methods & Protocols 6: bpab017. 10.1093/biomethods/bpab017 PMC8476930 34595352

[eva70333-bib-0031] Follak, S. , S. Dullinger , I. Kleinbauer , D. Moser , and F. Essl . 2013. “Invasion Dynamics of Three Allergenic Invasive Asteraceae ( *Ambrosia trifida* , *Artemisia annua* , *Iva xanthiifolia* ) in Central and Eastern Europe.” Preslia 85: 41–61.

[eva70333-bib-0032] Frankham, R. 1995. “Conservation Genetics.” Annual Review of Genetics 29: 305–327. 10.1146/annurev.ge.29.120195.001513 8825477

[eva70333-bib-0093] Genton, B. J. , J. A. Shykoff , and T. Giraud . 2005. “High Genetic Diversity in French Invasive Populations of Common Ragweed, *Ambrosia artemisiifolia*, as a Result of Multiple Sources of Introduction.” Molecular Ecology 14, no. 14: 4275–4285. 10.1111/j.1365-294x.2005.02750.x. 16313592

[eva70333-bib-0099] Glaubitz, J. C. , T. M. Casstevens , F. Lu , et al. 2014. “TASSEL‐GBS: A High Capacity Genotyping by Sequencing Analysis Pipeline.” PLoS One 9, no. 2: e90346. 10.1371/journal.pone.0090346. PMC3938676 24587335

[eva70333-bib-0033] Godsoe, W. 2010. “Regional Variation Exaggerates Ecological Divergence in Niche Models.” Systematic Biology 59: 298–306. 10.1093/sysbio/syq005 PMC2910897 20525637

[eva70333-bib-0034] Goplen, J. J. , C. C. Sheaffer , R. L. Becker , et al. 2016. “Giant Ragweed ( *Ambrosia trifida* ) Seed Production and Retention in Soybean and Field Margins.” Weed Technology 30: 246–253.

[eva70333-bib-0095] Gorton, A. J. , D. A. Moeller , and P. Tiffin . 2018. “Little Plant, Big City: A Test of Adaptation to Urban Environments in Common Ragweed (*Ambrosia artemisiifolia*).” Proceedings of the Royal Society B: Biological Sciences 285, no. 1881: 20180968. 10.1098/rspb.2018.0968. PMC6030533 30051853

[eva70333-bib-0035] Guisan, A. , B. Petitpierre , O. Broennimann , C. Daehler , and C. Kueffer . 2014. “Unifying Niche Shift Studies: Insights From Biological Invasions.” Trends in Ecology & Evolution 29: 260–269. 10.1016/j.tree.2014.02.009 24656621

[eva70333-bib-0036] Guisan, A. , and W. Thuiller . 2005. “Predicting Species Distribution: Offering More Than Simple Habitat Models.” Ecology Letters 8: 993–1009. 10.1111/j.1461-0248.2005.00792.x 34517687

[eva70333-bib-0037] Halsted, B. D. 1889. “Our Worst Weeds.” Botanical Gazette 14: 69–71.

[eva70333-bib-0038] Harrison, S. K. , E. E. Regnier , and J. T. Schmoll . 2003. “Postdispersal Predation of Giant Ragweed ( *Ambrosia trifida* ) Seed in No‐Tillage Corn.” Weed Science 51: 955–964.

[eva70333-bib-0039] Harrison, S. K. , E. E. Regnier , J. T. Schmoll , and J. E. Webb . 2001. “Competition and Fecundity of Giant Ragweed in Corn.” Weed Science 49: 224–229.

[eva70333-bib-0040] Hodgins, K. A. , P. Battlay , and D. G. Bock . 2025. “The Genomic Secrets of Invasive Plants.” New Phytologist 245: 1846–1863. 10.1111/nph.20368 39748162

[eva70333-bib-0041] Kalinowski, S. T. 2004. “Counting Alleles With Rarefaction: Private Alleles and Hierarchical Sampling Designs.” Conservation Genetics 5: 539–543.

[eva70333-bib-0042] Kalinowski, S. T. 2005. “HP‐RARE 1.0: A Computer Program for Performing Rarefaction on Measures of Allelic Richness.” Molecular Ecology Notes 5: 187–189.

[eva70333-bib-0043] Kato‐Noguchi, D. , and M. Kato . 2024. “Invasive Characteristics and Impacts of *Ambrosia trifida* .” Agronomy 14, no. 12: 2868.

[eva70333-bib-0044] Kil, J. H. , K. C. Shim , S. H. Park , et al. 2004. “Distributions of Naturalized Alien Plants in South Korea.” Weed Technology 18: 1493–1495.

[eva70333-bib-0045] Kim, C. G. , and J. Kil . 2016. “Alien Flora of the Korean Peninsula.” Biological Invasions 18: 1843–1852.

[eva70333-bib-0046] Kim, J. M. , Y. J. Im , and E. S. Jun . 2000. Naturalized Plants of Korea. Science Books.

[eva70333-bib-0048] Kim, K. D. 2017. “Distribution and Management of the Invasive Exotic Species *Ambrosia trifida* and *Sicyos angulatus* in the Seoul Metropolitan Area.” Journal of Ecological Engineering 18: 27–36.

[eva70333-bib-0049] Kimura, M. 1964. “Diffusion Models in Population Genetics.” Journal of Applied Probability 1: 177–232.

[eva70333-bib-0050] Kołodziejczyk, J. , A. Fijarczyk , I. Porth , et al. 2025. “Genomic Investigations of Successful Invasions: The Picture Emerging From Recent Studies.” Biological Reviews 100: 1396–1418. 10.1111/brv.70005 PMC12120398 39956989

[eva70333-bib-0051] Korres, N. E. , J. K. Norsworthy , M. V. Bagavathiannan , and A. Mauromoustakos . 2015. “Distribution of Arable Weed Populations Along Eastern Arkansas Mississippi Delta Roadsides: Occurrence, Distribution and Favored Growth Habitats.” Weed Technology 29: 587–595.

[eva70333-bib-0110] Krasovec, M. , M. Chester , K. Ridout , and D. A. Filatov . 2018. “The Mutation Rate and the Age of the Sex Chromosomes in *Silene latifolia* .” Current Biology 28, no. 11: 1832–1838. 10.1016/j.cub.2018.04.069. 29804812

[eva70333-bib-0052] Langmead, B. , and S. L. Salzberg . 2012. “Fast Gapped‐Read Alignment With Bowtie 2.” Nature Methods 9: 357–359. 10.1038/nmeth.1923 PMC3322381 22388286

[eva70333-bib-0053] Lee, C. E. 2002. “Evolutionary Genetics of Invasive Species.” Trends in Ecology & Evolution 17: 386–391.

[eva70333-bib-0054] Lee, C. S. , Y. C. Cho , H. C. Shin , G. S. Kim , and J. H. Pi . 2010. “Control of an Invasive Alien Species, *Ambrosia trifida* , With Restoration by Introducing Willows as Typical Riparian Vegetation.” Journal of Ecology and Field Biology 33: 157–164.

[eva70333-bib-0055] Lee, S.‐R. , T.‐Y. Choi , and D.‐C. Son . 2024. “Multiple Introductions of Divergent Lineages and Admixture Conferred High Invasiveness in a Widespread Weed ( *Hypochaeris radicata* ).” Evolutionary Applications 17: 1–16. 10.1111/eva.13740 PMC11192970 38911265

[eva70333-bib-0056] Lee, S.‐R. , and D. C. Son . 2022. “Genetic Diversity Pattern Reveals the Primary Determinant of Burcucumber ( *Sicyos angulatus* L.) Invasion in Korea.” Frontiers in Plant Science 13: 1–12. 10.3389/fpls.2022.997521 PMC9706109 36457533

[eva70333-bib-0057] Lee, W. T. , and H. P. Chong . 1976. “The Flora of Mt. Samak and Jungdo.” Korean Journal of Plant Taxonomy 7: 1–20.

[eva70333-bib-0058] Lee, Y. N. , and Y. J. Oh . 1974. “Naturalized Plants of Korea (I).” Journal of the Korean Research Institute for Better Living 12: 25–31.

[eva70333-bib-0096] Li, F. , M. van Kleunen , J. Li , et al. 2019. “Patterns of Genetic Variation Reflect Multiple Introductions and Pre‐Admixture Sources of Common Ragweed (*Ambrosia artemisiifolia*) in China.” Biological Invasions 21, no. 6: 2191–2209. 10.1007/s10530-019-01966-2.

[eva70333-bib-0059] Liu, C. , C. Wolter , W. Xian , and J. M. Jeschke . 2020. “Most Invasive Species Largely Conserve Their Climatic Niche.” Proceedings of the National Academy of Sciences of the United States of America 117: 23643–23651. 10.1073/pnas.2004289117 PMC7519298 32883880

[eva70333-bib-0109] Lynch, M. , and J. S. Conery . 2000. “The Evolutionary Fate and Consequences of Duplicate Genes.” Science 290, no. 5494: 1151–1155. 10.1126/science.290.5494.1151. 11073452

[eva70333-bib-0106] Mairal, M. , S. L. Chown , J. Shaw , et al. 2021. “Human Activity Strongly Influences Genetic Dynamics of the Most Widespread Invasive Plant in the Sub‐Antarctic.” Molecular Ecology 31, no. 6: 1649–1665. 10.1111/mec.16045. 34181792

[eva70333-bib-0060] McGaughran, A. , M. K. Dhami , E. Parvizi , et al. 2024. “Genomic Tools in Biological Invasions: Current State and Future Frontiers.” Genome Biology and Evolution 16: 1–16. 10.1093/gbe/evad230 PMC10776249 38109935

[eva70333-bib-0061] Mooney, H. A. , and E. E. Cleland . 2001. “The Evolutionary Impact of Invasive Species.” Proceedings of the National Academy of Sciences of the United States of America 98: 5446–5451. 10.1073/pnas.091093398 PMC33232 11344292

[eva70333-bib-0062] Novak, S. J. , and R. N. Mack . 2005. “Genetic Bottlenecks in Alien Plant Species.” In Species Invasions, edited by D. F. Sax , J. J. Stachowicz , and S. D. Gaines , 201–228. Sinauer Associates.

[eva70333-bib-0100] Peakall, R. , and P. E. Smouse . 2012. “GenAlEx 6.5: Genetic Analysis in Excel. Population Genetic Software for Teaching and Research—An Update.” Bioinformatics 28, no. 19: 2537–2539. 10.1093/bioinformatics/bts460. PMC3463245 22820204

[eva70333-bib-0063] Peterson, B. K. , J. N. Weber , E. H. Kay , H. S. Fisher , and H. E. Hoekstra . 2012. “Double Digest RADseq: An Inexpensive Method for De Novo SNP Discovery and Genotyping in Model and Non‐Model Species.” PLoS One 7: e37135. 10.1371/journal.pone.0037135 PMC3365034 22675423

[eva70333-bib-0064] Petitpierre, B. , C. Kueffer , O. Broennimann , C. Randin , C. Daehler , and A. Guisan . 2012. “Climatic Niche Shifts Are Rare Among Terrestrial Plant Invaders.” Science 335: 1344–1348. 10.1126/science.1215933 22422981

[eva70333-bib-0065] Pickrell, J. K. , and J. K. Pritchard . 2012. “Inference of Population Splits and Mixtures From Genome‐Wide Allele Frequency Data.” PLoS Genetics 8: e1002967. 10.1371/journal.pgen.1002967 PMC3499260 23166502

[eva70333-bib-0066] Pimentel, D. , L. Lach , R. Zuniga , D. Morrison , and D. Pimentel . 2000. “Environmental and Economic Costs of Nonindigenous Species in the United States.” Bioscience 50: 53–65.

[eva70333-bib-0097] Prapas, D. , R. Scalone , J. Lee , et al. 2022. “Quantitative Trait Loci Mapping Reveals an Oligogenic Architecture of a Rapidly Adapting Trait During the European Invasion of Common Ragweed.” Evolutionary Applications 15, no. 8: 1249–1263. 10.1111/eva.13453. PMC9423086 36051461

[eva70333-bib-0067] Prentis, P. J. , J. R. U. Wilson , E. E. Dormontt , D. M. Richardson , and A. J. Lowe . 2008. “Adaptive Evolution in Invasive Species.” Trends in Plant Science 13: 288–294. 10.1016/j.tplants.2008.03.004 18467157

[eva70333-bib-0068] Pritchard, J. K. , M. Stephens , and P. Donnelly . 2000. “Inference of Population Structure Using Multilocus Genotype Data.” Genetics 155: 945–959. 10.1093/genetics/155.2.945 PMC1461096 10835412

[eva70333-bib-0069] Purcell, S. , B. Neale , K. Todd‐Brown , et al. 2007. “PLINK: A Tool Set for Whole‐Genome Association and Population‐Based Linkage Analyses.” American Journal of Human Genetics 81: 559–575. 10.1086/519795 PMC1950838 17701901

[eva70333-bib-0070] Putra, A. R. , K. A. Hodgins , and A. Fournier‐Level . 2024. “Assessing the Invasive Potential of Different Source Populations of Ragweed ( *Ambrosia artemisiifolia* L.) Through Genomically Informed Species Distribution Modelling.” Evolutionary Applications 17: 1–13. 10.1111/eva.13632 PMC10810254 38283606

[eva70333-bib-0072] Qin, Z. , A. DiTommaso , R. S. Wu , and H. Y. Huang . 2014. “Potential Distribution of Two *Ambrosia* Species in China Under Projected Climate Change.” Weed Research 54: 520–531.

[eva70333-bib-0073] R Core Team . 2025. R: A Language and Environment for Statistical Computing. R Foundation for Statistical Computing.

[eva70333-bib-0074] Raj, A. , M. Stephens , and J. K. Pritchard . 2014. “fastSTRUCTURE: Variational Inference of Population Structure in Large SNP Data Sets.” Genetics 197: 573–589. 10.1534/genetics.114.164350 PMC4063916 24700103

[eva70333-bib-0075] Rasmussen, K. , J. Thyrring , R. Muscarella , and F. Borchsenius . 2017. “Climate‐Change‐Induced Range Shifts of Three Allergenic Ragweeds (*Ambrosia* L.) in Europe and Their Potential Impact on Human Health.” PeerJ 5: e3104. 10.7717/peerj.3104 PMC5357339 28321366

[eva70333-bib-0092] Regnier, E. , S. K. Harrison , J. Liu , et al. 2008. “Impact of an Exotic Earthworm on Seed Dispersal of an Indigenous US Weed.” Journal of Applied Ecology 45, no. 6: 1621–1629. 10.1111/j.1365-2664.2008.01489.x.

[eva70333-bib-0076] Regnier, E. E. , S. K. Harrison , M. M. Loux , et al. 2016. “Certified Crop Advisors' Perceptions of Giant Ragweed ( *Ambrosia trifida* ) Distribution, Herbicide Resistance and Management in the Corn Belt.” Weed Science 64: 361–377.

[eva70333-bib-0077] Rochette, N. C. , A. G. Rivera‐Colón , and J. M. Catchen . 2019. “Stacks 2: Analytical Methods for Paired‐End Sequencing Improve RADseq‐Based Population Genomics.” Molecular Ecology 28: 4737–4754. 10.1111/mec.15253 31550391

[eva70333-bib-0078] Rousset, F. 1997. “Genetic Differentiation and Estimation of Gene Flow From F‐Statistics Under Isolation by Distance.” Genetics 145: 1219–1228. 10.1093/genetics/145.4.1219 PMC1207888 9093870

[eva70333-bib-0079] Schutte, B. J. 2007. Biology and Ecology of *Ambrosia trifida* L. PhD Dissertation. Ohio State University.

[eva70333-bib-0080] Seebens, H. , S. Bacher , T. M. Blackburn , et al. 2021. “The Evolutionary Dynamics of Biological Invasions: A Multi‐Approach Perspective.” Evolutionary Applications 14: 1463–1484. 10.1111/eva.13215 PMC8210789 34178098

[eva70333-bib-0081] Sherpa, S. , and L. Després . 2021. “The Evolutionary Dynamics of Biological Invasions: A Multi‐Approach Perspective.” Evolutionary Applications 14: 1463–1484. 10.1111/eva.13215 PMC8210789 34178098

[eva70333-bib-0105] Simberloff, D. 2009. “The Role of Propagule Pressure in Biological Invasions.” Annual Review of Ecology, Evolution, and Systematics 40, no. 1: 81–102. 10.1146/annurev.ecolsys.110308.120304.

[eva70333-bib-0083] Stoller, E. W. , and L. M. Wax . 1974. “Dormancy Changes and Fate of Some Annual Weed Seeds in the Soil.” Weed Science 22: 151–155.

[eva70333-bib-0084] Václavík, T. , and R. K. Meentemeyer . 2012. “Equilibrium or not? Modelling Potential Distribution of Invasive Species in Different Stages of Invasion.” Diversity and Distributions 18: 73–83.

[eva70333-bib-0085] Vallejo‐Marín, M. , J. Friedman , A. D. Twyford , et al. 2021. “Population Genomic and Historical Analysis Suggests a Global Invasion by Bridgehead Processes in *Mimulus guttatus* .” Communications Biology 4: 1–12. 10.1038/s42003-021-01795-x PMC7954805 33712659

[eva70333-bib-0086] Van Boheemen, L. A. , E. Lombaert , K. A. Nurkowski , B. Gauffre , L. H. Rieseberg , and K. A. Hodgins . 2017. “Multiple Introductions, Admixture and Bridgehead Invasion Characterize the Introduction History of *Ambrosia artemisiifolia* .” Molecular Ecology 26: 5421–5434. 10.1111/mec.14293 28802079

[eva70333-bib-0087] Vitousek, P. M. , C. M. D'Antonio , L. L. Loope , and R. Westbrooks . 1996. “Biological Invasions as Global Environmental Change.” American Scientist 84: 468–478.

[eva70333-bib-0088] Wang, P. , W. Liang , C. Kong , and Y. Jiang . 2005. “Allelopathic Potential of Volatile Allelochemicals of *Ambrosia trifida* L. on Other Plants.” Allelopathy Journal 15: 131–136.

[eva70333-bib-0101] Warren, D. L. , R. E. Glor , and M. Turelli . 2008. “Environmental Niche Equivalency Versus Conservatism: Quantitative Approaches to Niche Evolution.” Evolution 62, no. 11: 2868–2883. 10.1111/j.1558-5646.2008.00482.x. 18752605

[eva70333-bib-0102] Warren, D. L. , R. E. Glor , and M. Turelli . 2010. “ENMTools: A Toolbox for Comparative Studies of Environmental Niche Models.” Ecography 33, no. 3: 607–611. 10.1111/j.1600-0587.2009.06142.x.

[eva70333-bib-0089] Washitani, I. 2001. “Plant Conservation Ecology for Management and Restoration of Riparian Habitats of Lowland Japan.” Population Ecology 43: 189–195.

[eva70333-bib-0090] Wilson, J. R. U. , E. E. Dormontt , P. J. Prentis , A. J. Lowe , and D. M. Richardson . 2009. “Something in the Way You Move: Dispersal Pathways Affect Invasion Success.” Trends in Ecology & Evolution 24: 136–144. 10.1016/j.tree.2008.10.007 19178981

[eva70333-bib-0091] Wortman, S. E. , A. S. Davis , B. J. Schutte , et al. 2012. “Local Conditions, not Regional Gradients, Drive Demographic Variation of Giant Ragweed ( *Ambrosia trifida* ) and Common Sunflower ( *Helianthus annuus* ) Across the Northern U.S. Maize Belt.” Weed Science 60: 440–450.

