# Supplementary material for: Repeated Introductions and Anthropogenic Dispersal Fuel the Regional Expansion of Ambrosia trifida
Source: Evol Appl. 2026 Oct 21;19(10):e70333. doi: 10.1111/eva.70333 (PMC13685678; doi:10.1111/eva.70333)
Supplement: Supplementary file 1 [file EVA-19-e70333-s001.docx]

Supplementary Information

Repeated introductions and anthropogenic dispersal fuel the regional expansion of *Ambrosia trifida*

Table S1. Comparison of genetic diversity estimates obtained using the original global minor allele frequency (MAF) filtering strategy and the alternative filtering strategy in which the MAF threshold was applied separately to the native US samples. Estimates of genetic diversity were highly consistent between the two filtering approaches, indicating that the principal conclusions of the study are robust to the SNP filtering strategy.

| Population acronym | Global MAF filter data | | | Local MAF filter data | | | |  |
| --- | --- | --- | --- | --- | --- | --- | --- | --- |
|  | Ho | | He | | Ho | | He | |
| **AS** | 0.23[0.16] | 0.30[0.15] | | 0.2[0.003] | | 0.27[0.003] | |  |
| **BD** | 0.25[0.18] | 0.27[0.16] | | 0.2[0.003] | | 0.23[0.003] | |  |
| **BF** | 0.18[0.15] | 0.24[0.15] | | 0.2[0.003] | | 0.25[0.003] | |  |
| **BY** | 0.16[0.14] | 0.24[0.16] | | 0.18[0.003] | | 0.23[0.003] | |  |
| **DD** | 0.17[0.18] | 0.18[0.13] | | 0.16[0.003] | | 0.18[0.003] | |  |
| **DG** | 0.26[0.19] | 0.33[0.14] | | 0.15[0.003] | | 0.19[0.003] | |  |
| **DJ** | 0.19[0.17] | 0.22[0.14] | | 0.16[0.003] | | 0.23[0.003] | |  |
| **GB** | 0.24[0.17] | 0.28[0.15] | | 0.14[0.003] | | 0.18[0.003] | |  |
| **GF** | 0.26[0.20] | 0.27[0.17] | | 0.17[0.003] | | 0.19[0.003] | |  |
| **GJ** | 0.26[0.17] | 0.29[0.14] | | 0.23[0.003] | | 0.26[0.003] | |  |
| **GR** | 0.24[0.18] | 0.27[0.15] | | 0.16[0.003] | | 0.18[0.003] | |  |
| **GS** | 0.26[0.18] | 0.30[0.15] | | 0.2[0.003] | | 0.22[0.003] | |  |
| **HN** | 0.22[0.15] | 0.29[0.14] | | 0.2[0.003] | | 0.28[0.003] | |  |
| **HS** | 0.29[0.21] | 0.39[0.12] | | 0.18[0.004] | | 0.2[0.003] | |  |
| **IC** | 0.23[0.15] | 0.30[0.14] | | 0.2[0.003] | | 0.27[0.003] | |  |
| **IF** | 0.20[0.17] | 0.25[0.15] | | 0.15[0.003] | | 0.23[0.003] | |  |
| **IM** | 0.25[0.19] | 0.26[0.16] | | 0.17[0.003] | | 0.18[0.003] | |  |
| **IO** | 0.28[0.18] | 0.31[0.14] | | 0.21[0.004] | | 0.22[0.003] | |  |
| **IS** | 0.28[0.21] | 0.29[0.15] | | 0.16[0.004] | | 0.16[0.003] | |  |
| **IT** | 0.30[0.20] | 0.35[0.13] | | 0.22[0.004] | | 0.22[0.003] | |  |
| **KS** | 0.25[0.17] | 0.32[0.16] | | 0.17[0.003] | | 0.22[0.003] | |  |
| **MC** | 0.24[0.17] | 0.29[0.14] | | 0.2[0.003] | | 0.27[0.003] | |  |
| **MG** | 0.23[0.19] | 0.27[0.16] | | 0.14[0.003] | | 0.2[0.003] | |  |
| **MI** | 0.23[0.18] | 0.28[0.14] | | 0.2[0.003] | | 0.25[0.003] | |  |
| **MY** | 0.26[0.20] | 0.27[0.15] | | 0.18[0.004] | | 0.19[0.003] | |  |
| **PF** | 0.27[0.19] | 0.36[0.13] | | 0.2[0.004] | | 0.22[0.003] | |  |
| **PS** | 0.30[0.21] | 0.35[0.13] | | 0.2[0.004] | | 0.21[0.003] | |  |
| **SA** | 0.25[0.17] | 0.31[0.15] | | 0.18[0.003] | | 0.24[0.003] | |  |
| **SC** | 0.24[0.18] | 0.26[0.16] | | 0.19[0.003] | | 0.22[0.003] | |  |
| **SW** | 0.24[0.17] | 0.31[0.15] | | 0.2[0.003] | | 0.27[0.003] | |  |
| **US** | 0.25[0.16] | 0.31[0.15] | | 0.21[0.003] | | 0.27[0.003] | |  |
| **YC** | 0.23[0.16] | 0.31[0.14] | | 0.18[0.003] | | 0.25[0.003] | |  |
| **YE** | 0.23[0.16] | 0.28[0.14] | | 0.2[0.003] | | 0.27[0.003] | |  |
| **YJ** | 0.21[0.12] | 0.29[0.13] | | 0.19[0.002] | | 0.29[0.002] | |  |
| **YW** | 0.22[0.17] | 0.27[0.14] | | 0.2[0.003] | | 0.25[0.003] | |  |
| **US1** | 0.21[0.15] | 0.26[0.15] | | 0.18[0.003] | | 0.25[0.003] | |  |
| **US2** | 0.16[0.14] | 0.23[0.14] | | 0.18[0.003] | | 0.26[0.003] | |  |
| **US3** | 0.20[0.15] | 0.26[0.14] | | 0.19[0.003] | | 0.24[0.003] | |  |
| **US4** | 0.18[0.15] | 0.22[0.15] | | 0.2[0.003] | | 0.25[0.003] | |  |

Table S2. Model selection results from Approximate Bayesian Computation (ABC) analyses for *Ambrosia trifida*. Seven alternative demographic scenarios were evaluated, and support for each scenario is shown as the proportion of votes obtained from the ABC model selection procedure. The posterior probability, global error rate, and local error rate are reported for the best-supported scenario, indicating the confidence and reliability of scenario discrimination.

| Scenarios | 1 | 2 | 3 | 4 | 5 | 6 | 7 |
| --- | --- | --- | --- | --- | --- | --- | --- |
| Votes | 0.000 | 0.006 | 0.002 | 0.000 | 0.857 | 0.065 | 0.070 |
| Posterior probability |  |  |  |  | 0.853 |  |  |
| Global error |  |  |  |  | 0.308 |  |  |
| Local error |  |  |  |  | 0.106 |  |  |

Table S3. Akaike Information Criterion (AIC) values for three demographic models fitted using FASTSIMCOAL2 for the three *Ambrosia trifida* demes identified by fastSTRUCTURE, used for demographic model comparison.

| Deme | Model 1 | Model 2 | Model 3 |
| --- | --- | --- | --- |
| 1 | 132.863 | 100.66 | 85.372 |
| 2 | 128.910 | 97.604 | 91.322 |
| 3 | 110.352 | 90.892 | 82.122 |

Figure S1. Historical changes in effective population size (Ne) of three *Ambrosia trifida* demes identified by fastSTRUCTURE and inferred using simulation-based demographic analyses implemented in FASTSIMCOAL2. Model parameters include N_ANC_ (ancestral effective population size), N_CON_ (contemporary effective population size), N_BOT_ (Bottlenecked effective population size), T_BOT1_ (time of bottleneck initiation), and T_BOT2_ (time of bottleneck termination).

Linearized FST

| Log of geographic distance | Y |
| --- | --- |
| 5.05134 | 0.158539 |
| 5.54364 | 0.120138 |
| 5.26279 | 0.149253 |
| 4.1012 | 0.118736 |
| 5.24448 | 0.159902 |
| 5.47368 | 0.0388841 |
| 4.81574 | 0.282999 |
| 5.22894 | 0.298365 |
| 5.8562 | 0.159115 |
| 5.19737 | 0.17344 |
| 5.11863 | 0.175065 |
| 4.91008 | 0.220314 |
| 4.49453 | 0.12729 |
| 5.03015 | 0.140681 |
| 5.58195 | 0.21552 |
| 3.99665 | 0.0972914 |
| 4.98804 | 0.155286 |
| 5.33082 | 0.0803291 |
| 3.80897 | 0.0710264 |
| 5.15143 | 0.158065 |
| 4.77495 | 0.11896 |
| 5.50742 | 0.332555 |
| 5.29411 | 0.356468 |
| 3.18757 | 0.237057 |
| 5.41401 | 0.256047 |
| 5.84645 | 0.375917 |
| 4.40489 | 0.37386 |
| 5.27678 | 0.295113 |
| 5.41588 | 0.228583 |
| 5.15681 | 0.3074 |
| 5.90162 | 0.256669 |
| 5.64508 | 0.262774 |
| 4.93358 | 0.418814 |
| 5.70837 | 0.323657 |
| 5.54481 | 0.230867 |
| 5.91649 | 0.498683 |
| 5.3794 | 0.078951 |
| 4.8402 | 0.110652 |
| 4.24372 | 0.0771968 |
| 5.3717 | 0.0917032 |
| 5.68646 | 0.233432 |
| 4.2481 | 0.134207 |
| 5.16898 | 0.0857254 |
| 4.40435 | 0.267262 |
| 5.69946 | 0.201195 |
| 5.27632 | 0.33465 |
| 4.46005 | 0.386574 |
| 5.61912 | 0.298748 |
| 5.49923 | 0.317691 |
| 5.11627 | 0.399372 |
| 5.39735 | 0.379339 |
| 5.33399 | 0.258567 |
| 5.6472 | 0.309977 |
| 4.56599 | 0.485207 |
| 5.33509 | 0.28991 |
| 5.37308 | 0.146279 |
| 5.06415 | 0.207345 |
| 5.85868 | 0.140565 |
| 5.6086 | 0.145152 |
| 4.9196 | 0.296753 |
| 5.65834 | 0.23118 |
| 5.49852 | 0.145697 |
| 5.87465 | 0.332626 |
| 2.80324 | 0.219651 |
| 5.64637 | 0.128805 |
| 4.39308 | 0.393552 |
| 4.67502 | 0.0514234 |
| 5.11661 | 0.124861 |
| 5.79384 | 0.0864392 |
| 5.11123 | 0.0913429 |
| 3.09878 | 0.259002 |
| 5.50007 | 0.13038 |
| 5.03967 | 0.0730898 |
| 5.78392 | 0.278275 |
| 4.92352 | 0.195306 |
| 5.61213 | 0.0497128 |
| 5.03046 | 0.288071 |
| 4.89189 | 0.123474 |
| 4.03969 | 0.202952 |
| 5.22944 | 0.225155 |
| 5.73513 | 0.125825 |
| 4.65728 | 0.141698 |
| 4.40157 | 0.178589 |
| 5.39967 | 0.186313 |
| 4.70173 | 0.11406 |
| 5.70848 | 0.370116 |
| 5.33375 | 0.376576 |
| 5.5857 | 0.141302 |
| 5.31374 | 0.333013 |
| 5.30421 | 0.227023 |
| 4.28643 | 0.154119 |
| 4.51085 | 0.0621028 |
| 5.30261 | 0.116572 |
| 5.82682 | 0.0811758 |
| 4.94974 | 0.0871253 |
| 3.99105 | 0.240999 |
| 5.52863 | 0.115944 |
| 4.97209 | 0.0669463 |
| 5.80648 | 0.29638 |
| 5.24557 | 0.194517 |
| 5.67816 | 0.0445864 |
| 5.30872 | 0.285152 |
| 5.22631 | 0.125459 |
| 3.99066 | 0.0236576 |
| 3.60667 | 0.132301 |
| 5.08153 | 0.113971 |
| 4.84679 | 0.164885 |
| 5.7546 | 0.112287 |
| 5.38654 | 0.108281 |
| 4.55196 | 0.214137 |
| 5.49954 | 0.14013 |
| 5.25388 | 0.0893697 |
| 5.76355 | 0.348616 |
| 4.18527 | 0.241565 |
| 5.52758 | 0.094906 |
| 4.34058 | 0.342157 |
| 4.01651 | 0.162021 |
| 4.44793 | 0.0789099 |
| 5.00408 | 0.155569 |
| 4.92855 | 0.0739235 |
| 4.82602 | 0.230238 |
| 5.30665 | 0.272497 |
| 5.21752 | 0.216918 |
| 4.31206 | 0.236962 |
| 5.50877 | 0.364262 |
| 4.71765 | 0.298409 |
| 4.35129 | 0.210269 |
| 5.1158 | 0.47755 |
| 5.80192 | 0.40848 |
| 5.19741 | 0.190464 |
| 5.61419 | 0.429596 |
| 5.76448 | 0.299431 |
| 5.43623 | 0.198605 |
| 5.17363 | 0.308149 |
| 5.35807 | 0.183547 |
| 5.58597 | 0.249696 |
| 5.27724 | 0.19432 |
| 4.95777 | 0.207623 |
| 5.81135 | 0.144647 |
| 5.53271 | 0.161742 |
| 4.81236 | 0.183279 |
| 5.59268 | 0.174565 |
| 5.41261 | 0.130966 |
| 5.82587 | 0.344034 |
| 3.53133 | 0.310881 |
| 5.58997 | 0.138311 |
| 4.26172 | 0.200085 |
| 3.04985 | 0.208255 |
| 4.76306 | 0.160044 |
| 5.21129 | 0.126805 |
| 5.13694 | 0.147039 |
| 3.58474 | 0.166541 |
| 5.69948 | 0.25317 |
| 5.15286 | 0.508026 |
| 4.73665 | 0.476327 |
| 5.72136 | 0.348783 |
| 5.43068 | 0.373981 |
| 4.77246 | 0.340878 |
| 5.47592 | 0.485984 |
| 5.28598 | 0.369775 |
| 5.73586 | 0.49009 |
| 4.1541 | 0.644794 |
| 5.47859 | 0.376075 |
| 3.97698 | 0.560799 |
| 3.90495 | 0.49346 |
| 4.67708 | 0.455293 |
| 5.1194 | 0.40502 |
| 5.07171 | 0.455806 |
| 3.21787 | 0.449728 |
| 5.60248 | 0.576566 |
| 3.42102 | 0.290045 |
| 5.24896 | 0.156035 |
| 5.00618 | 0.186617 |
| 5.8298 | 0.113374 |
| 5.51454 | 0.123718 |
| 4.70726 | 0.169451 |
| 5.60626 | 0.126859 |
| 5.40118 | 0.0925461 |
| 5.84154 | 0.343045 |
| 3.57294 | 0.293837 |
| 5.61584 | 0.110068 |
| 4.41774 | 0.241575 |
| 3.33063 | 0.18197 |
| 4.66631 | 0.125413 |
| 5.16097 | 0.118672 |
| 5.07058 | 0.107621 |
| 3.44591 | 0.125031 |
| 5.69379 | 0.24296 |
| 2.7178 | 0.0543416 |
| 3.59259 | 0.332724 |
| 5.2228 | 0.163743 |
| 4.91225 | 0.217307 |
| 4.27756 | 0.161656 |
| 5.15864 | 0.176012 |
| 5.63146 | 0.351343 |
| 3.10745 | 0.194509 |
| 4.92981 | 0.169464 |
| 4.23022 | 0.389452 |
| 5.72302 | 0.335181 |
| 3.9168 | 0.127085 |

Figure S2. Relationship between geographic distance and pairwise genetic differentiation (F_ST_) among 35 Korean populations of *Ambrosia trifida*. Isolation by distance was evaluated using a Mantel test based on linearized F_ST_ values and Euclidean geographic distances, and no significant correlation was detected (r = 0.10, P > 0.05).

a)

PC2 (4.4%)

| PC1 (6.1%) | US1 |
| --- | --- |
| -0.164102 | -3.86817 |
| 0.584087 | 0.297326 |
| 1.0161 | -2.90713 |
| -3.26051 | 2.55769 |
| 2.07323 | -2.13704 |
| -11.0031 | 3.76589 |
| 1.51472 | -2.80094 |
| -7.46104 | 5.08591 |
| -7.31544 | 5.0079 |
| 3.19719 | 0.00733383 |
| 1.5772 | -1.03641 |
| -10.4763 | 4.23228 |
| -3.92247 | -8.3854 |
| -3.1841 | 3.88997 |
| 0.330211 | -3.99729 |

| PC1 (6.1%) | US2 |
| --- | --- |
| -1.50966 | -3.89623 |
| 3.23775 | -0.99893 |
| 3.37069 | 1.13459 |
| -5.91224 | 6.37095 |
| 2.09009 | -1.41479 |
| 0.863569 | 5.17856 |
| 4.38509 | 1.23742 |
| -0.338579 | 4.42791 |
| 4.44937 | 1.13221 |
| 2.14086 | 2.28645 |
| -0.00413371 | 2.99807 |
| -2.23506 | 0.228198 |
| -6.70158 | 1.81872 |
| 4.06375 | 0.348012 |

| PC1 (6.1%) | US3 |
| --- | --- |
| 3.70376 | 0.229482 |
| -2.21817 | -8.70502 |
| -2.38267 | 5.26174 |
| -3.08396 | -9.00248 |
| -5.94766 | -10.3964 |
| 0.127538 | -6.67496 |
| -4.99113 | -9.99923 |
| -0.757699 | -6.66983 |
| -4.88596 | -10.0002 |
| 1.71878 | -2.54846 |
| -4.61825 | -1.44628 |
| 0.542395 | -4.51312 |
| -2.12976 | 2.06291 |
| 0.72177 | -5.97519 |
| 0.253162 | 2.26576 |

| PC1 (6.1%) | US4 |
| --- | --- |
| 3.38921 | 0.661939 |
| -8.10045 | 4.46285 |
| -1.70746 | 0.679157 |
| -7.08589 | 6.14319 |
| 4.77592 | 1.93564 |
| 4.37014 | 0.664267 |
| -1.55542 | 2.93834 |
| 4.54747 | 1.32147 |
| -3.72238 | 3.44194 |
| 1.89315 | -2.44991 |
| -1.95362 | 4.08868 |
| 3.29894 | 0.649868 |
| -9.30925 | 5.19019 |
| 4.68198 | 1.41061 |
| -2.54809 | 1.80143 |

| PC1 (6.1%) | KR |
| --- | --- |
| 3.30571 | 0.829647 |
| -0.83798 | -2.8575 |
| 4.82342 | 0.935526 |
| 3.73634 | -0.463497 |
| 2.93957 | -0.841543 |
| 4.25941 | 0.290975 |
| 4.6631 | 0.659331 |
| -7.04364 | -2.65495 |
| 3.48795 | 0.517068 |
| 3.40909 | 0.209253 |
| 3.80051 | 0.716505 |
| 3.67375 | 0.789572 |
| 3.11531 | -0.352235 |
| 3.97754 | 1.03494 |
| -3.32306 | -1.2455 |
| 0.469475 | -2.58013 |
| 4.36481 | 1.1724 |
| -2.93747 | 2.67683 |
| 2.38383 | 0.515016 |
| -3.96188 | -4.32173 |
| -3.85383 | -0.518667 |
| 2.0803 | 0.776391 |
| -1.61379 | 2.15631 |
| -1.55933 | 6.20448 |
| 1.78126 | 2.33236 |
| 4.32902 | 0.40836 |
| 4.88266 | 0.393179 |
| 3.63379 | 0.886369 |
| 4.85718 | 0.863607 |
| 2.88849 | 0.447774 |
| 2.20738 | 2.16619 |
| -2.3456 | 5.78918 |
| 0.411957 | -4.33302 |
| 4.21508 | 0.682894 |
| -5.62972 | 3.31716 |
| 4.43852 | 0.919237 |
| -7.69562 | -3.05877 |
| -2.7352 | -0.517058 |
| -1.03012 | -0.20252 |
| 3.07598 | 0.211985 |
| 2.33322 | -0.914334 |
| 4.73033 | 1.43034 |
| 3.9841 | 0.712447 |
| 1.51719 | 0.182654 |
| 2.09285 | 1.30522 |
| 4.27016 | 0.559436 |
| 2.22763 | -1.17425 |
| 2.9614 | 0.811367 |
| 2.78865 | 0.712957 |
| -8.16975 | -3.12076 |
| 0.732221 | -2.48046 |
| 1.21316 | 1.48484 |
| -1.60865 | -0.359862 |
| -8.52737 | -2.74357 |
| -4.87968 | 3.48086 |
| -2.12296 | -3.53283 |
| 3.25603 | 1.04226 |
| 3.96311 | 0.799404 |
| 4.16218 | 0.457447 |

PC2 (4.3%)

| PC1 (5.6%) | US1 |
| --- | --- |
| -1.07679 | -3.35339 |
| -0.138052 | 1.04964 |
| 0.0516827 | -2.68548 |
| -3.84809 | 3.1331 |
| 0.998314 | -1.91224 |
| -11.5431 | 4.22554 |
| 0.554844 | -2.36481 |
| -7.84156 | 5.33104 |
| -7.43297 | 5.34233 |
| 2.30868 | 0.278998 |
| 0.807595 | -0.762628 |
| -10.8648 | 4.61982 |
| -4.80488 | -7.76063 |
| -3.92975 | 4.22681 |
| -0.485191 | -3.6997 |

| PC1 (5.6%) | US2 |
| --- | --- |
| -2.38199 | -3.45698 |
| 2.51272 | -0.581338 |
| 2.58152 | 1.41232 |
| -6.46735 | 6.59183 |
| 1.19119 | -1.11828 |
| 0.360812 | 5.31586 |
| 3.85994 | 1.48159 |
| -0.874389 | 5.06765 |
| 3.82179 | 1.37701 |
| 1.63182 | 3.33401 |
| -0.634975 | 3.60848 |
| -2.77553 | 0.863502 |
| -7.47807 | 2.76669 |
| 3.36697 | 0.458002 |

| PC1 (5.6%) | US3 |
| --- | --- |
| 3.14216 | 0.259726 |
| -3.19217 | -8.48725 |
| -2.65651 | 5.17085 |
| -3.67203 | -8.75243 |
| -6.81853 | -10.0063 |
| -0.730582 | -6.54697 |
| -5.92219 | -9.5816 |
| -1.66642 | -6.77485 |
| -5.72486 | -9.50804 |
| 0.932231 | -2.43848 |
| -4.89688 | -0.941939 |
| -0.0736865 | -4.33304 |
| -2.46895 | 2.25595 |
| -0.120861 | -5.92998 |
| -0.515754 | 2.59666 |

| PC1 (5.6%) | US4 |
| --- | --- |
| 2.42231 | 0.872583 |
| -8.64173 | 5.05428 |
| -2.47126 | 1.2339 |
| -7.38573 | 6.41716 |
| 4.20031 | 2.68557 |
| 3.62943 | 0.889986 |
| -2.04394 | 3.26974 |
| 3.93397 | 1.63561 |
| -4.33902 | 3.95995 |
| 1.02758 | -2.17918 |
| -2.40946 | 4.2986 |
| 2.46491 | 1.17261 |
| -9.88911 | 5.63072 |
| 3.98979 | 2.0915 |
| -2.9696 | 2.7349 |

| PC1 (5.6%) | KR |
| --- | --- |
| 4.97025 | 0.387693 |
| -0.0524343 | -4.40043 |
| 3.12944 | 1.05919 |
| 2.33276 | -0.532666 |
| 4.09112 | 2.01033 |
| -1.42547 | -5.81711 |
| 1.40422 | 1.03568 |
| -2.11815 | -2.80418 |
| 4.29683 | 0.469666 |
| 2.94056 | 0.386701 |
| 1.42858 | 2.35362 |
| 3.59361 | 1.12099 |
| 2.03903 | 1.57724 |
| 0.0724282 | -5.35719 |
| 4.05993 | 0.991963 |
| 2.1755 | -0.351344 |
| 3.90597 | 0.645596 |
| 2.84948 | 0.223328 |
| 2.21817 | -0.0965331 |
| -3.81341 | -0.777115 |
| -0.710565 | -2.39324 |
| 3.95769 | 0.118069 |
| -3.5524 | -2.05929 |
| 4.57538 | 0.579967 |
| 1.32827 | -0.00751133 |
| 3.34652 | 1.14007 |
| 0.251107 | 0.0568306 |
| 3.50687 | 0.693037 |
| 4.1108 | 1.14622 |
| 3.50331 | 0.949193 |
| 1.45888 | 2.93606 |
| 4.13473 | 0.763021 |
| 2.14294 | -2.78596 |
| 3.78813 | 1.63318 |
| 3.39545 | 1.27202 |
| 3.42556 | 0.24037 |
| 4.36414 | 0.795635 |
| 3.39789 | 0.514087 |
| -3.46073 | -0.83512 |
| 2.43973 | -1.13599 |
| 3.59121 | 1.413 |
| -3.68302 | 0.00365712 |
| 3.53828 | 0.906663 |
| -4.70864 | -1.08526 |
| 2.35186 | -1.68386 |
| 3.45622 | 0.591691 |
| -5.57684 | -1.74217 |
| 4.39216 | 0.290226 |
| 2.31628 | 0.405651 |
| 3.50497 | 1.11181 |
| 3.90994 | 0.519286 |
| -5.08836 | -1.21017 |
| 3.96389 | 1.27783 |
| 1.25041 | -0.00317987 |
| -0.649725 | -2.01868 |
| -2.68185 | -0.337756 |
| 0.666063 | -3.68618 |
| 4.36797 | -0.13862 |
| 2.9733 | 0.101081 |

PC2 (4.4%)

| PC1 (6.2%) | US1 |
| --- | --- |
| -0.172715 | -3.42325 |
| 0.417903 | 0.4023 |
| 0.880342 | -2.52025 |
| -3.23343 | 2.72546 |
| 1.84062 | -1.74419 |
| -11.1126 | 4.04865 |
| 1.39292 | -2.33157 |
| -7.66712 | 5.54241 |
| -7.35522 | 5.56089 |
| 3.06023 | 0.308421 |
| 1.30631 | -0.709396 |
| -10.7557 | 4.54698 |
| -4.03652 | -8.08389 |
| -3.21081 | 4.28677 |
| 0.15769 | -3.59797 |

| PC1 (6.2%) | US2 |
| --- | --- |
| -1.65255 | -3.63735 |
| 3.32526 | -0.586123 |
| 3.15831 | 1.31279 |
| -6.08966 | 6.69613 |
| 2.02384 | -1.04413 |
| 0.757433 | 5.282 |
| 4.14274 | 1.25776 |
| -0.35516 | 4.406 |
| 4.3997 | 1.17187 |
| 2.32403 | 2.2743 |
| -0.244336 | 2.82381 |
| -2.27187 | 0.0112762 |
| -6.77701 | 1.46882 |
| 3.89483 | 0.451076 |

| PC1 (6.2%) | US3 |
| --- | --- |
| 3.52174 | 0.371697 |
| -2.50909 | -8.56642 |
| -2.62542 | 5.46617 |
| -3.06657 | -8.96081 |
| -6.2174 | -10.3864 |
| -0.0645624 | -6.33916 |
| -5.2265 | -9.89108 |
| -0.99327 | -6.59122 |
| -4.98898 | -9.75978 |
| 1.57174 | -2.45413 |
| -4.52568 | -1.74226 |
| 0.228441 | -4.25992 |
| -2.38456 | 2.05102 |
| 0.459551 | -5.65986 |
| 0.0754328 | 2.28369 |

| PC1 (6.2%) | US4 |
| --- | --- |
| 3.12187 | 0.847082 |
| -8.21484 | 4.49132 |
| -1.82089 | 0.60979 |
| -7.261 | 6.30697 |
| 4.77144 | 2.11147 |
| 4.29911 | 0.850181 |
| -1.58703 | 2.88277 |
| 4.36321 | 1.35981 |
| -3.80013 | 3.30309 |
| 1.79193 | -2.2211 |
| -2.15584 | 4.29042 |
| 3.26668 | 0.876982 |
| -9.52916 | 5.21283 |
| 4.69782 | 1.5996 |
| -2.42807 | 1.43626 |

| PC1 (6.2%) | KR |
| --- | --- |
| 3.07908 | 0.204004 |
| 1.27562 | -1.09534 |
| -4.65537 | 3.52781 |
| 2.08289 | 1.60962 |
| 2.33592 | -1.98191 |
| -6.11138 | -2.14966 |
| 2.64895 | 0.469311 |
| -2.68307 | 3.78234 |
| 4.19532 | 0.388018 |
| -5.20268 | -2.88826 |
| 2.86074 | 0.801566 |
| 2.18327 | 0.7392 |
| -1.86746 | -1.68634 |
| 2.75699 | 2.40427 |
| -10.8423 | -1.89387 |
| 1.82704 | 1.48568 |
| 4.09688 | 0.959821 |
| -1.7211 | -4.15737 |
| 2.79411 | 0.445993 |
| 4.38409 | 0.838139 |
| -5.09009 | 4.9193 |
| 2.14752 | -1.13857 |
| 3.03857 | -1.04275 |
| -2.60577 | 5.75883 |
| 3.47076 | -0.252581 |
| -8.67774 | -3.98387 |
| 3.12015 | 0.768605 |
| 3.79714 | 0.584793 |
| 1.76447 | 2.50711 |
| 2.10316 | -1.06329 |
| 3.83903 | 0.764598 |
| -1.01486 | -1.65113 |
| 3.18433 | -0.399673 |
| 2.05536 | 1.27199 |
| -4.23694 | -3.24253 |
| 3.78933 | -0.207866 |
| -4.68254 | -3.78158 |
| 3.56173 | 0.137433 |
| 4.78077 | 0.560224 |
| 3.17981 | -1.13319 |
| 4.42422 | 0.756668 |
| -1.46899 | 2.40378 |
| 3.3168 | -0.219897 |
| 2.75092 | -1.63419 |
| 4.26957 | 0.598625 |
| 3.43945 | 1.03729 |
| 3.23302 | 0.869607 |
| -3.91145 | -4.34193 |
| 4.94293 | 0.659088 |
| 4.02586 | 0.642799 |
| -1.65013 | -0.743968 |
| 3.06337 | 0.738915 |
| 4.48454 | 1.00785 |
| 3.10361 | 0.802595 |
| 3.14384 | -0.00765264 |
| -0.424737 | -2.66659 |
| 4.69497 | 0.558954 |
| 1.86263 | 1.37282 |
| 4.82034 | 0.567815 |

b)

Figure S3. Sensitivity analyses using downsampled introduced samples. To evaluate the potential effect of unequal sample sizes between native (n = 59) and introduced (n = 419) samples, ten independent downsampling replicates were generated by randomly selecting 59 Korean individuals to match the native sample size. (a) Three representative principal coordinate analysis (PCoA) plots from the ten downsampling replicates. Similar to the full dataset, the downsampled datasets showed diffuse clustering and substantial overlap among individuals. (b) fastSTRUCTURE bar plots (K = 3) for all ten downsampling replicates. Most replicates recovered genetic assignment patterns comparable to those obtained from the full dataset, indicating that the inferred population structure is robust to sample-size imbalance.

a)

b)

Figure S4. Sensitivity analyses using an alternative minor allele frequency (MAF) filtering strategy. To evaluate the potential effect of global MAF filtering on the representation of native-range variation, the MAF threshold was applied separately to the native US samples, and the resulting dataset was compared with the original globally filtered dataset. (a) Principal coordinate analysis (PCoA) of the alternatively filtered dataset. Similar to the original dataset, the ordination showed diffuse clustering and substantial overlap among individuals. (b) fastSTRUCTURE bar plots based on the alternatively filtered dataset. The overall genetic assignment patterns were comparable to those obtained using the original global MAF filtering strategy, indicating that the inferred population structure was robust to the SNP filtering approach.

a)

b)

Figure S5. Bayesian clustering analysis of *A*. *trifida* inferred using fastSTRUCTURE for K = 2-10. (a) Ancestry proportions for 35 Korean (KR) populations. (b) Ancestry proportions for the complete dataset including both 35 Korean and four native US populations. Each vertical bar represents an individual, with colors indicating proportional membership in inferred genetic clusters. Populations are separated by dashed white lines and labeled according to the population codes in Table 1.

Figure S6. Model selection for TreeMix analysis based on the number of migration edges (m). The plot shows changes in log-likelihood as a function of m (0–5). Change points were evaluated using piecewise linear, bent-cable, and simple exponential models to identify the optimal number of migration events. Allowing a single migration edge (m = 1) resulted in the largest improvement in model fit, after which log-likelihood values plateaued.

Figure S7. Density distributions of 19 bioclimatic variables in native (green) and introduced (red) environmental spaces for *Ambrosia trifida*. Shaded areas represent occurrence densities, while solid and dashed lines indicate the available environmental background in each range. Blue arrows denote the direction and magnitude of niche shifts between native and introduced ranges.

Figure S8. Linear discriminant analysis (LDA) plot projecting simulated training datasets and the observed dataset onto the first two LDA axes based on summary statistics for the selected demographic scenario.

|  |  |
| --- | --- |
| FST1m_3 | 27278500000 |
| LDA1 | 15350200000 |
| FST1v_1 | 10604500000 |
| FST1m_1 | 5301490000 |
| HBm_2.4 | 5013680000 |
| FST1v_3 | 4896500000 |
| HBm_2.3 | 2976770000 |
| Comp 1 | 1995320000 |
| HBm_1.4 | 1840340000 |
| Comp 3 | 1120320000 |
| FST1v_2 | 1074360000 |
| F3m_1.2.3 | 1041130000 |
| HBm_3.4 | 885421000 |
| HWv_1 | 873959000 |
| F3m_3.1.2 | 671063000 |
| F3v_1.2.3 | 542199000 |
| Comp 6 | 531843000 |
| HBm_1.2 | 453244000 |
| FST1v_4 | 451283000 |
| ML3p_1.3.4 | 397674000 |
| HBm_1.3 | 390434000 |
| AMLm_2.1.4 | 344512000 |
| AMLm_1.2.3 | 300131000 |
| F3m_2.1.3 | 295196000 |
| F3m_4.1.2 | 287923000 |
| Comp 2 | 254840000 |
| AMLv_1.2.3 | 243709000 |
| AMLm_1.3.4 | 211782000 |
| AMLm_3.1.2 | 209777000 |
| F3m_3.2.4 | 179498000 |

Figure S9. Relative contribution of the 30 most informative summary statistics to the random forest used for model selection among the nine demographic scenarios.
